# Dietary CP and amino acid restriction has a different impact on the dynamics of protein, amino acid and fat deposition in entire male, castrated and female pigs

**DOI:** 10.1017/S1751731118000770

**Published:** 2018-05-23

**Authors:** I. Ruiz-Ascacibar, P. Stoll, M. Kreuzer, G. Bee

**Affiliations:** 1 Agroscope, La Tioleyre 4, 1725 Posieux, Switzerland; 2 Institute of Agricultural Sciences, ETH Zurich, Universitätsstrasse 2, 8092 Zurich, Switzerland

**Keywords:** amino acid profile, allometric equations, fat and protein relative deposition, amino acid relative deposition, dietary protein deficiency

## Abstract

Breeding efforts over the last decades altered markedly empty body (EB) composition of pigs. This study aimed to re-evaluate the dynamics of changes in the composition and deposition rate of fat, protein and amino acids (AA) in the EB from birth to 140 kg BW depending on the dietary CP and AA supply in a current pig genotype. In the experiment 66 entire male, 58 castrated and 66 female Swiss Large White pigs were used. From 20 kg BW onwards, they had either *ad libitum* access to a control (C) diet or a diet (LP) compared to diet C only 80% of CP, lysine, methione+cystine, threonine and tryptophan. The EB composition was determined at birth on eight boars and eight females, at 10 and 20 kg BW on two boars, two castrates and two females, and at 20 kg intervals from 40 to 140 kg BW, on four pigs per gender and dietary treatment. Each EB fraction was weighed and analysed for protein, fat and AA profile. The AA-to-lysine ratio was calculated and the different chemical component contents were fitted to allometric regressions. Overall, C-boars had the greatest EB protein and AA content and deposition rates, and lowest fat content and deposition rates. At the beginning of the grower period, LP-castrates and females displayed the lowest protein and AA and the highest fat deposition rates. However, compared with their counterparts in the C-group, in LP-castrates and females protein and AA deposition rates were greater above 64 and 40 kg EB weight, respectively, whereas fat deposition rates was lower above 80 kg EB weight. Thus, there seems a great potential to optimise protein and AA efficiency especially in the finisher period in castrates and females. Important individual variations were found in the essential AA-to-lysine ratio of the EB. Phenylalanine and threonine-to-lysine ratios decreased with increasing EB weight. Valine- and threonine-to-lysine ratios in C-castrates and C-females were 5% and 4% greater than recently reported by the National Research Council (NRC) whereas cysteine-, methionine- and tyrosine-to-lysine ratios were lower by 34%, 25% and 10%, respectively. The clear differences found between the EB AA-to-lysine ratios in the present study and the NRC might partly be explained by the genotype and the temporal changes in the relative weight of each EB fraction or changes in the AA profile. Nevertheless, these findings on changes in the essential AA profile of tissue protein warrant further studies.

## Implications

The equations for the amount of empty body (EB) protein, amino acid (AA) and fat obtained from this data set can be used for future updates on nutrient deposition rates of modern pig genotypes. The present study reports changes in the amount and relative deposition rates of AA from birth to 140 kg BW. The large variation found in the AA profile and the influence of BW on the phenylalanine-, threonine and histidine-to-lysine ratios questions the steadiness of the whole EB AA profile.

## Introduction

Growth models of pigs, which are primarily based on whole body protein and fat deposition results (Nieto *et al*., 2012), are valuable tools to optimise the efficiency in pig production since they are used to elaborate, evaluate and refine feeding recommendations for grower-finisher pigs. One of the primary breeding goals of the last decades was the selection of lean carcasses (Brocks *et al*., 2000) which has resulted in marked changes in the body’s chemical composition and nutrient deposition rates. Current recommendations for protein and AA requirements rely on the ideal protein concept, based on studies of the pig body’s AA composition of, at least, 19 years ago (i.e. current version of National Research Council (NRC), 2012). However, some studies have questioned the use of a constant AA profile. For instance, Mahan and Shields (1998) suggested that the ideal essential amino acid (EAA) ratio changes with pig weight, age and genotype due to changes of the carcass protein deposition and the decrease of the proportion of the non-carcass protein component, which have different AA profiles. Along the same line, Conde-Aguilera *et al*. (2010) showed that the AA composition of the proteins found in different tissues of piglets changed with the supply of dietary sulphur-containing AA. The serial slaughter technique is laborious and expensive but it is also the only method delivering reliable data on macro- and micronutrient composition of the EB (Schinckel *et al*., 2008; Ruiz-Ascacibar *et al*., 2017). We recently reported the daily protein and fat deposition in pigs of different gender and BW depending on dietary CP supply (Ruiz-Ascacibar *et al*., 2017). To extend the information on the dynamics of compositional changes of the EB, the present evaluation aimed at using the allometric model to describe relative fat, protein and AA deposition rates from birth to 140 kg as a function of the empty BW (EBW). Since differences in the AA profile of the pig’s EB were expected due to changes in the relative proportion of the different body components over the last decades, gender and CP and AA supply, the second objective was to re-evaluate the AA-to-lysine ratio in the EB of a modern lean pig genotype like the Swiss Large White breed.

## Material and methods

The experimental procedures were approved by the Swiss Cantonal Committee for Animal Care and Use (2012-14-FR 22119 and 2013-24-FR 24064).

### Animals, diets and experimental design

A total of 190 pigs originating from four farrowing groups, 66 females (FE), 58 castrates (CA) and 66 entire males (EM), from the Agroscope sow herd were used in the experiment. In farrowing round one and two 106 Premo^®^×Large White crossbred pigs (serie 1) and farrowing round three and four 84 Swiss Large White×Swiss Large White pigs (serie 2) were included. A detailed description of the management protocol and the composition of the experimental diets have been previously reported by Ruiz-Ascacibar *et al*. (2017). On the day of birth eight FE and eight EM weighing 1.4±0.44 (mean±SD) were slaughtered. The next slaughter events took place at 8.9±0.20 kg (mean±SD) with two FE, two CA and two EM, and at 20.8±1.66 with eight FE, eight CA and eight EM. The remaining 144 pigs were allotted per gender, to one of the six subsequent slaughter weight categories (40, 60, 80, 100, 120 or 140 kg BW) and assigned to either a control (C) or a low CP (LP) diet. The grower (20 to 60 kg BW), finisher I (60 to 100 kg BW) and finisher II diets (100 to 140 kg BW) of group C were formulated according to the Swiss standard feeding recommendations for pigs weighing 40, 80 and 120 kg BW, respectively (Agroscope, 2017), whereas the grower, finisher I and finisher II diets of the LP-group were formulated to contain only 80% of dietary digestible CP, lysine, methionine+cystine, threonine and tryptophan as compared with the corresponding C-diets. In both dietary treatments, EM were fed diets with 5% greater amounts of digestible CP, lysine, methionine+cystine, threonine and tryptophan than CA and FE to account for their greater protein deposition potential (Agroscope, 2017). All C and LP diets were designed to be isocaloric (13.2 MJ digestible energy/kg). When the individual pig reached the initial BW defined for the grower, finisher I and finisher II period (BW >19, 59 and 98 kg, respectively), it was allocated to the corresponding diet. The analysed composition of the experimental diets has been published in detail in Ruiz-Ascacibar *et al*. (2017).

### Slaughter procedure and sampling

The comparative slaughter procedure was used to determine the protein, fat and AA amounts of the EB of the pigs. A detailed description of the slaughter methods, sampling and grinding were previously presented (Ruiz-Ascacibar *et al*., 2017). In brief, when the individual pigs reached their target BW for slaughter, they were fasted for ~16 h, stunned with CO_2_ and exsanguinated. To determine the chemical composition of the EB of each pig, one representative sample of the following five fractions was obtained, which had been weighed before: (I) carcass (including head, tail and feet), (II) viscera and intestine (including heart, kidneys, liver, lungs, tongue, spleen, eyes, brain, ear, mesentery, belly fat, empty bladder, empty gallbladder, blood, stomach, large intestine and hindgut), (III) blood, (IV) skin and claws and (V) bile. All subsamples were stored at −20°C until being lyophilised (Christ, delta, Newtown, UK) for 70 h.

### Chemical analyses

Separate aliquots of the different body parts as well as diets were analysed for dry matter content and CP. Dry matter in feed was determined by gravimetry, after drying at 105°C for 3 h. The CP (total N×6.25) amount was analysed with a LECO FP-2000 analyser (Leco, Mönchengladbach, Germany) (International Organization for Standardization (ISO), 2008). Amino acid analyses in lyophilised samples were performed according to Baumann *et al*. (2010). For the AA analyses in lyophilised samples, an aliquot of 1 to 2 mg was hydrolysed with 6 m HCl for 22 h at 115°C according to Chang and Knecht (1991). The hydrolysated AA were labelled with phenylisothiocyanate and the resulting phenylthiocarbamil derivates were separated and quantified by reversed-HPLC (Dionex Summit HPLC System, Thermo Fisher Scientific, Reinach, Switzerland).

### Calculations and statistical analysis

The sum of the five portions (blood, hair and hooves, organs and intestines, carcass and bile) was considered as the entire EB. The data regarding the relative weight of the carcass, organs and intestine and blood were fitted to EBW using an allometric regression (Supplementary Table S1). Data on EB were calculated from proportionate wet weights and chemical composition of each of these parts. Protein, fat and AA amounts of EB were calculated from the proportionate wet weights, the chemical composition and the wet weights of each of the five fractions. The AA profile of the EB was expressed as g AA/100 g lysine. Significant differences in the EBW between diets and among genders, within each slaughter weight category were observed when data were first analysed. This might explain the general lack of normally distributed data. Therefore, a pre-treatment of the data was necessary. In brief, data on fat, protein and AA amount of the EB were corrected using the allometric model with standardized EBW categories. A data pre-treatment is described in detail in Supplementary Material S1. These corrected values will be referred to as transformed data of EB protein, AA and fat amounts. Ranks were assigned to the transformed data from 40 to 140 kg and the ranked data were subjected to the ANOVA procedure of SYSTAT 13 (SYSTAT Software, Inc.) considering the experimental groups (C-EM, C-FE, C-CA, LP-EM, LP-FE, LP-CA), serie and the two- and three-way interactions as fixed effects. None of the interactions with BW were significant, therefore BW factor was removed and ranks were then assigned to the transformed data within each BW category from 40 to 140 kg. These ranked data were then subjected to the ANOVA procedure of SYSTAT 13 (SYSTAT Software, Inc.) considering the experimental groups (C-EM, C-FE, C-CA, LP-EM, LP-FE, LP-CA), serie and the two-way interaction as fixed effects. To assess whether any of the diet×gender combinations could be plotted in the same regression equation, the following predefined orthogonal contrasts were tested:

∙ FE *v*. CA fed either diet C or LP
  ◦ C-FE *v*. C-CA
  ◦ LP-FE *v*. LP-CA
∙ EM *v*. FE/CA fed either diet C or LP
  ◦ C-EM *v*. C-FE/CA
  ◦ LP-EM *v*. LP-FE/CA
∙ Diet C *v*. diet LP regardless of gender
  ◦ C-FE/CA/EM *v*. LP-FE/CA/EM

Based on the outcome of the orthogonal contrast, the untransformed data of protein, AA and fat amount of the EB were grouped and fitted using the untransformed data and the non-linear procedure of SYSTAT 13, to the following allometric regression; *Y*=*a*×EBW^*b*^, where *Y* is the predicted EB protein, AA or fat amount; *b* the scaling exponent or allometric coefficient and *a* the constant. The EB relative protein, AA and fat deposition rates, expressed in g/kg EBW gain, were calculated as the first derivative of the aforementioned allometric function for each EBW (*Y*′=*a*×*b*×EBW^(*b*−1)^). The lysine-to-protein and the relative AA-to-lysine deposition ratios were estimated from the lysine, relative protein and AA deposition rates calculated with the first derivative of the allometric regression. Ranks were also assigned to the AA-to-lysine ratios of the EB and they were subsequently subjected to the ANOVA procedure of SYSTAT 13 (SYSTAT Software, Inc.) considering the dietary treatment, gender, BW category, the serie as well as their corresponding interaction as fixed effects.

## Results

### Realised diet composition

Diet composition was previously detailed by Ruiz-Ascacibar *et al*. (2017). The intended reduction by 20% from C- to LP-diets in the amounts of apparent ileal digestible CP and apparent ileal digestible lysine, methionine+cystine, threonine, tryptophan was successfully achieved. However, the ileal digestible isoleucine-to-lysine ratio of the grower- and finisher II LP-diets was 0.53 and therefore 0.1 unit lower compared with the Swiss feeding recommendations for pigs (Agroscope, 2017).

### Average amounts of protein, amino acids and fat of the empty body

The average amount of protein, fat and EAA in the EB found in FE, CA and EM across all BW categories from 40 to 140 kg are presented separately for the C- and LP-group in Table 1. The individual means for the different BW categories and for the periods where all animals were fed the standard weaner diets are listed in Supplementary Table S2. The amount of EB protein, threonine, phenylalanine, valine, leucine and isoleucine in the LP-group was lower (*P*<0.05) on average compared with group C (Table 1). However, in group C but not LP, the average amount of EB protein, threonine, phenylalanine, valine, leucine and isoleucine was greater (*P*<0.05) in EM than FE and CA. Overall, the amount of lysine was lower (*P*<0.05) in the LP- than C-group. Furthermore, in the C-group the amount of lysine was lower (*P*<0.05) whereas in the LP-group the amount of lysine was greater (*P*<0.05) in FE and CA than EM. Methionine, methionine+cystine, tyrosine and histidine amounts were lower (*P*<0.05) in the LP-group compared with the C-group. The cystine amount of the EB was similar within dietary treatments and gender. Overall, the EB fat amount was greater (*P*<0.05) in the LP-group compared with the C-group and decreased within dietary treatment from CA>FE>EM.

**Table 1:** Protein, amino acids and fat content^1^ of the empty body (g) from female (FE), castrated (CA) and entire male (EM) pigs from 40 to 140 kg BW fed either a control (C) or low CP (LP) diet,^2^ estimated across the BW categories of 40, 60, 80, 100, 120 and 140 kg

	Control			Low CP			Orthogonal
Diet	EM	CA	FE	EM	CA	FE	contrasts^3^
Protein	15 077 (1221.3)	14 324 (1095.0)	14 420 (1112.7)	14 068 (1137.0)	14 367 (1171.9)	14 152 (1140.5)	c, e
Fat	14 188 (1585.3)	19 233 (2393.2)	17 573 (2206.3)	17 261 (1936.4)	21 337 (2392.4)	19 277 (2087.4)	a, b, c, d, e
Amino acid							
Lysine	1 051 (91.9)	938 (70.6)	956 (74.3)	754 (60.7)	932 (76.3)	947 (83.0)	c, d, e
Methionine	215 (23.8)	193 (18.8)	208 (17.2)	183 (17.0)	185 (15.6)	199 (22.1)	e
Cystine	93 (8.9)	88 (7.6)	83 (6.7)	78 (7.6)	85 (8.3)	86 (9.8)	–
Methionine+cystine	309 (31.8)	281 (25.4)	290 (23.0)	261 (23.4)	269 (23.2)	285 (30.5)	e
Threonine	583 (49.7)	522 (37.1)	522 (42.9)	511 (42.7)	498 (39.6)	506 (43.4)	c, e
Phenylalanine	541 (45.5)	492 (37.0)	488 (37.0)	473 (39.6)	473 (37.5)	479 (41.7)	c, e
Tyrosine	359 (33.8)	323 (25.4)	323 (26.7)	310 (27.0)	318 (26.6)	320 (28.0)	e
Valine	730 (62.5)	653 (48.1)	652 (49.2)	635 (53.8)	634 (51.0)	644 (57.3)	c, e
Leucine	1 050 (90.4)	959 (72.0)	956 (75.0)	931 (80.0)	929 (75.6)	942 (84.7)	c, e
Isoleucine	539 (49.4)	484 (37.7)	491 (40.8)	470 (41.5)	473 (39.6)	481 (44.3)	c, e
Histidine	473 (43.8)	439 (41.0)	459 (42.6)	439 (39.4)	427 (36.8)	427 (39.4)	e

To assess whether any of the diet×gender combinations could be plotted in the same regression equation, data (ranks were assigned to the transformed data within each BW category from 40 to 140 kg) were subjected to the ANOVA procedure considering the experimental groups (C-EM, C-FE, C-CA, LP-EM, LP-FE, LP-CA), period and the two-way interaction as fixed effects, followed by orthogonal contrast.

1 Results are expressed as means (standard deviations) of the fat, protein and amino acid content of the transformed data (detailed description in Supplementary Material S1). These transformed data correspond to an average empty BW of 87 (6.9) kg.

2 The C-diets were formulated to meet nutrient requirements for grower-finisher pigs in the grower, finisher I and finisher II periods according to the Swiss feeding recommendations for pigs; the LP-diets were formulated to contain, expressed as percentage of C-diets, 80% of dietary CP, lysine, methionine+cystine, threonine and tryptophan.

3 Significant (*P*<0.05) orthogonal contrast were expressed by a letter as follows: a=C-FE *v*. C-CA; b=LP-FE *v*. LP-CA; c=C-EM *v*. C-FE/CA; d=LP-EM *v*. LP-FE/CA; e=C-FE/CA/EM *v*. LP-FE/CA/EM.

### Predicted empty body protein amount and relative deposition rate

**Figure 1:**
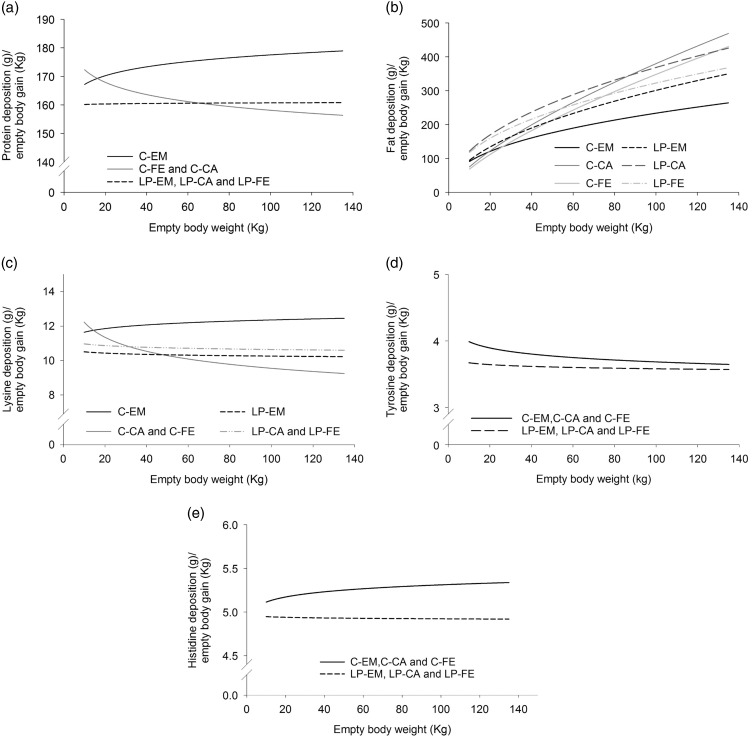
Dynamics of changes in relative deposition rates (g/kg empty BW gain) of protein (a), fat (b), lysine (c), tyrosine (d) and histidine (e) with increasing empty BW, grouped on the basis of orthogonal contrasts between genders and diets as specified and tested in Table 1. Data of pigs from birth to 140 kg BW were used for calculating the allometric equations. EM=entire males; CA=castrated pigs; FE=females pigs. The control diets (C) were formulated to meet nutrient requirements according to the standard Swiss feeding recommendations for grower-finisher pigs in the respective growth periods; the low CP diets (LP) were formulated to contain, expressed as percentage of the C-diets, 80% of dietary CP, lysine, methionine+cystine, threonine and tryptophan.

The allometric regression coefficients and curves determined for the protein amount of the EB of growing-finishing pigs, grouped based on the previously reported dietary treatments and gender differences, are presented in Table 2. The *b* coefficients for EB protein amount were close to 1 for all treatment groups indicating an almost linear increase in the protein amount with increasing EBW (Table 2). However, small differences were observed among the groups because C-EM and LP-EM, LP-FE and LP-CA displayed b coefficients greater than 1 whereas the *b* coefficient for C-FE and C-CA was slightly below 1. Empty body relative protein deposition rates, estimated from the first derivative of the allometric function, increased with increasing EBW in C-EM, decreased with increasing EBW in C-FE and C-CA and remained almost constant over the whole growth period in LP-EM, LP-FE and LP-CA (Figure 1a). Above 65 kg EBW, protein relative deposition rates were greater in LP-FE and LP-CA than C-FE and C-CA (Figure 1a).

**Table 2:** Allometric growth coefficients^1^ for protein, fat and amino acid amount (g) of the empty body of female (FE), castrated (CA) and entire male (EM) pigs from birth to 140 kg BW fed the control (C) or low CP (LP) diets^2^

Item	Diet	Gender	*a*	ASE of *a*	*b*	ASE of *b*	*R* ^2^
Protein	C	EM	153.4	17.66	1.026	0.0248	0.993
		CA, FE	195.0	16.18	0.963	0.0179	0.991
	LP	EM, CA, FE	159.3	8.85	1.002	0.0119	0.994
Fat	C	EM	25.3	9.22	1.409	0.0776	0.967
		CA	8.8	3.24	1.703	0.0778	0.977
		FE	7.9	3.33	1.705	0.0885	0.970
	LP	EM	19.9	5.37	1.502	0.0570	0.984
		CA	27.1	6.59	1.481	0.0513	0.986
		FE	30.0	5.14	1.437	0.0363	0.993
Amino acids							
Lysine	C	EM	10.7	3.55	1.026	0.0715	0.944
		CA, FE	17.5	3.83	0.893	0.0474	0.928
	LP	EM	10.9	2.64	0.990	0.0522	0.967
		CA, FE	11.5	2.37	0.987	0.0445	0.947
Methionine	C	EM, CA, FE	3.4	1.17	0.919	0.0750	0.788
	LP	EM, CA, FE	2.5	0.69	0.977	0.0585	0.872
Cystine	C, LP	EM, CA, FE	1.1	0.29	0.974	0.0561	0.803
Methionine+cystine	C	EM, CA, FE	5.1	1.55	0.906	0.0658	0.824
	LP	EM, CA, FE	3.1	0.76	1.008	0.0524	0.901
Threonine	C	EM	6.0	1.74	1.023	0.0624	0.957
		CA, FE	10.2	2.38	0.881	0.0507	0.916
	LP	EM, CA, FE	7.9	1.38	0.931	0.0375	0.938
Phenylalanine	C	EM	6.0	1.76	1.008	0.0636	0.954
		CA, FE	9.4	1.95	0.885	0.0450	0.933
	LP	EM, CA, FE	7.0	1.12	0.944	0.0346	0.948
Tyrosine	C	EM, CA, FE	4.5	1.04	0.966	0.0504	0.902
	LP	EM, CA, FE	3.8	0.63	0.989	0.0356	0.950
Valine	C	EM	7.5	2.29	1.023	0.0654	0.952
		CA, FE	13.3	2.70	0.872	0.0442	0.934
	LP	EM, CA, FE	9.0	1.50	0.954	0.0359	0.945
Leucine	C	EM	10.7	3.32	1.025	0.0668	0.951
		CA, FE	16.4	3.20	0.910	0.0423	0.944
	LP	EM, CA, FE	11.9	1.89	0.977	0.0341	0.953
Isoleucine	C	EM	4.8	1.95	1.055	0.0871	0.924
		CA, FE	8.3	2.10	0.911	0.0548	0.910
	LP	EM, CA, FE	5.5	0.94	1.000	0.0369	0.948
Histidine	C	EM, CA, FE	4.8	1.33	1.016	0.0591	0.884
	LP	EM, CA, FE	5.0	1.05	0.998	0.0455	0.924

ASE=asymptotic standard error. The different treatments (C-EM, C-CA, C-FE, LP-EM, LP-CA and LP-FE) were grouped based on the outcome of the orthogonal contrasts between genders and diets, as specified and tested in Table 1.

1 The allometric regression used was *Y*=*a*×EBW^*b*^, where *Y* is the predicted amount (g) of EB protein, amino acid or fat; EBW the empty BW (kg); *b* the allometric coefficient; *a* the constant. *R* ^2^ is based on the original and untransformed data.

2 The C-diets were formulated to meet nutrient requirements for grower-finisher pigs in the grower, finisher I and finisher II periods according to the Swiss feeding recommendations for pigs; the LP-diets were formulated to contain, expressed as percentage of C-diets, 80% of dietary CP, lysine, methionine+cystine, threonine and tryptophan.

### Predicted amino acid amount and relative deposition rate in the empty body

The allometric regression coefficients determined for the amounts for EAA of the EB and separately for each of the six treatments are presented in the Supplementary Table S3. The allometric regression coefficients determined for the amounts of the EAA of the EB in the grower, finisher I and finisher II period grouped according to the outcome of the orthogonal test are displayed in Table 2. Except for cystine, methionine and the sum of cystine+methionine, where the *R* ^2^ of the regression equation ranged from 0.79 to 0.90, the *R* ^2^ of the allometric regression of the other AA was greater than 0.9. The *b* coefficients determined for the EB amount of the nine EAA analysed in the present study ranged from 0.872 to 1.055 (Table 2). Values slightly above 1 were observed in C-EM for lysine, threonine, phenylalanine, valine, leucine, isoleucine, in C-pigs for histidine and in LP-pigs for methionine+cystine and isoleucine (Table 2). In all other experimental groups the *b* coefficients were below 1, indicating a slight decline in the amount with increasing EBW. In accordance, the relative deposition rates of lysine, methionine, threonine, phenylalanine, valine and leucine in both, C-FE and C-CA as well as in LP-FE, LP-CA and LP-EM decreased with increasing EBW. However, LP-pigs displayed greater relative deposition rates than C-CA and C-FE pigs above an approximate EBW of 40 kg (Figure 1c).

### Lysine-to-protein and amino acid-to-lysine ratios

The lysine amount of the protein and the AA profile, expressed as AA-to-lysine ratios, in the EB at different BW of FE, CA and EM pigs fed the standard weaner diets and subsequently either the C- or LP-diets are displayed in the Supplementary Figure S1. Additionally, the relative weight in the EB and the AA-to-lysine ratios of the carcass, organs and intestines, blood and skin and claws fractions, are presented in the Supplementary Tables S4 to S7, respectively. Regardless of the dietary treatments, the AA-to-lysine ratio was highly variable among individuals. Except for the phenylalanine-to-lysine and threonine-to-lysine ratios which decreased (*P*<0.05) with increasing BW, and the histidine-to-lysine ratio which increased (*P*<0.05) with the increasing BW, the AA profile was unaffected by the dietary treatments, gender and BW category (Table 3 and Supplementary Figure S1).

**Table 3:** Lysine to protein (g/100 g protein) and amino acid profile (g amino acid/100 g lysine)^1^ in the empty body of female (FE), castrated (CA) and entire male (EM) pigs fed the control (C) or low CP (LP) grower (20 to 60 kg), finisher I (60 to 100 kg) and finisher II (100 to 140 kg) diets^2^

	Diet C^2^			Diet LP^2^			*P*-value^3^		
Items	EM	CA	FE	EM	CA	FE	Diet	Gender	BW category
Lysine to protein	7.0 (0.2)	6.7 (0.19)	6.7 (0.22)	6.4 (0.14)	6.8 (0.22)	6.7 (0.22)	0.299	0.906	0.710
Methionine	20.7 (1.17)	20.4 (1.14)	21.7 (0.75)	20.3 (1.00)	22.3 (0.57)	21.3 (0.96)	0.824	0.658	0.522
Cystine	9.1 (0.50)	9.5 (0.56)	8.9 (0.59)	8.6 (0.45)	8.9 (0.35)	9.1 (0.5.0)	0.994	0.398	0.656
Methionine +cystine	29.8 (1.44)	30.0 (1.45)	30.5 (1.09)	28.9 (1.14)	31.3 (0.78)	30.3 (1.18)	0.962	0.598	0.803
Threonine	55.6 (0.53)	56.1 (1.07)	54.4 (0.64)	56.3 (1.19)	53.9 (0.67)	54.9 (1.01)	0.360	0.068	0.014
Phenylalanine	52.0 (0.59)	52.5 (0.56)	51.3 (0.60)	52.2 (0.79)	51.2 (0.52)	52.0 (0.66)	0.562	0.843	0.012
Tyrosine	33.9 (0.65)	34.3 (0.63)	33.6 (0.44)	34.1 (0.77)	34.0 (0.52)	34.6 (0.61)	0.288	0.910	0.731
Valine	69.8 (0.66)	69.9 (0.83)	68.6 (0.94)	69.9 (1.09)	68.3 (0.70)	69.6 (1.03)	0.904	0.698	0.584
Leucine	100.3 (0.61)	102.4 (1.61)	100.2 (1.05)	101.9 (1.15)	99.8 (0.78)	101.4 (1.02)	0.635	0.955	0.794
Isoleucine	51.0 (0.54)	51.4 (0.68)	51.0 (0.50)	51.1 (0.64)	50.5 (0.42)	51.6 (0.52)	0.771	0.569	0.236
Histidine	44.4 (0.79)	45.5 (1.72)	47.0 (1.29)	47.8 (1.75)	45.4 (0.94)	45.7 (1.19)	0.963	0.688	<0.001

1 Values are expressed as least square means across the slaughter BW categories of 40, 60, 80, 100, 120 and 140 kg. Standard error of the least square means are displayed in brackets.

2 The C-diets were formulated to meet nutrient requirements for grower-finisher pigs in the grower, finisher I and finisher II periods according to the Swiss feeding recommendations for pigs; the LP-diets were formulated to contain, expressed as percentage of C-diets, 80% of dietary CP, lysine, methionine+cystine, threonine and tryptophan.

3 Probability values for the effects of diet, gender and BW category. The probability values for the effect of serie and the interactions are not shown.

### Predicted empty body fat amount and relative deposition rate

The allometric coefficients for the EB fat amount were greater than 1.4 in all treatment groups, pointing to a curvilinear increase in the EB fat amount as pigs get heavier (Table 2). In the C-group, the lowest *b* coefficients were found in EM and the highest in CA, with intermediate values for FE. By contrast, among LP-pigs, LP-EM displayed the greatest *b* coefficients, LP-FE the lowest values and LP-CA had intermediate values. Despite the lower allometric coefficients, EB fat content was greater in LP-CA and LP-FE than in their C-counterparts. However, above 75 and 87 kg EBW relative fat deposition rate of C-FE and C-CA pigs surpassed relative fat deposition rate of LP-FE and LP-CA, respectively (Figure 1b). This can be explained by the lower *a*-value in FE and CA of treatment C (Table 2). Control-EM deposited less fat than LP-pigs at all times.

## Discussion

### Suitability of the allometric model used

Allometric equations have been widely used to model the dynamics of EB protein and fat accretion in relation to changes in BW (de Lange *et al*., 2003; Schinckel *et al*., 2008; Kouba and Bonneau, 2009). In the present study we confirmed their suitability for describing the dynamics of relative nutrient deposition. The *R* ^2^ ranging, from 0.79 to 0.99, demonstrates the excellent goodness of fit with the experimental data on changes in the EB protein, fat and EAA amount. Furthermore, the first derivative of these functions allowed to easily determine the dynamics of the relative protein, AA and fat deposition rates per unit EB change.

### Dynamics of protein and fat amount and relative deposition rates in the empty body

The data indicate that the LP-diet did not allow the EM to realise their full growth potential. This was even exacerbated by the inadequate supply of specific EAA. Low-CP grower and finisher II diets of period I, as well as LP-EM diets of period II had an unbalanced EAA pattern. For example the ratio of ileal digestible isoleucine-to-lysine was lower (0.53) than the one proposed by the Swiss feeding recommendations for pigs (0.63) (Agroscope, 2017). Consequently, the detrimental effects of the LP-diet on the EM could also be due to their inability to cope with the unbalanced AA profile of the aforementioned diets. In earlier studies, similar findings obtained with females or castrated pigs were reported showing that decreasing dietary CP supply below feeding recommendations lowers lean tissue and increase fat deposition rate (Kerr and Easter, 1995; Wood *et al*., 2013). Differences in the dynamics of relative fat and protein deposition in C-CA and C-FE and their LP counterparts indicate that, above an EBW of 75 kg (for fat deposition) and 65 kg (for protein deposition), dietary CP requirements of these pigs are below the current recommendations for optimal tissue deposition. Interestingly this occurred despite the EAA imbalances of the finisher LP-diets.

The present results on EB protein content and relative deposition rates of C-CA and C-FE were, on average, 18% greater than in the study of Schinckel *et al*. (2008) on high lean-gain pigs. However, predicted EB fat amount at 115 kg EBW in C-FE and C-CA was 18% and 15% greater, respectively, compared with the EB fat amount in the high lean-gain females and castrates in the study of Schinckel *et al*. (2008). In fact, relative fat deposition rate of the high lean-gain barrows and gilts were closer to what was observed in the EM of the present study. The differences described in the amount of EB fat and relative deposition rates in females and castrates between the present study and that of Schinckel *et al*. (2008) could be attributed to either nutrition or the interval of BW studied or the genotype.

The EB protein content of C-CA and C-FE in the present study was only 4% greater than that estimated using the quadratic equation proposed by the German Society of Nutrition Physiology (Gesellschaft für Ernährungsphysiologie (GfE), 2006). However, relative protein deposition rate calculated according to GfE (2006) declined much faster than what was observed in the present study for C-FE and C-CA pigs, and was, above 40 kg EBW, lower than the relative protein deposition rate for LP-pigs. Using the GfE (2006) equation, amount of EB fat was 45% greater than what was observed in C-FE and C-CA but similar to the amount of EB fat of LP-CA.

The greater estimate of the EB fat amount using the GfE (2006) equation could be attributed not only to the diet and gender, but also to the wide range of breeds, differing in their overall leanness (Large White, Pietrain, Yorksire, Cotswold, Duroc, etc.), included in the data set and to the quite old data (1983 to 2004) that were used, which do not account for the breeding progress in leanness of pigs occurring in the meantime.

### Dynamics of the amino acid amounts and relative deposition rates in the empty body

The relative AA deposition rate of C-EM was steady and greater from ~30 kg EBW onwards. The relative AA deposition rate diminished with increasing EBW in C-CA and C-FE pigs while above of ~40 kg EBW, relative AA deposition rate was greater in the LP-group than in C-CA and C-FE. This further supports the aforementioned hypothesis that LP-diets did not provide sufficient dietary CP and AA for EM pigs. In contrast, for CA and FE pigs the current Swiss feeding recommendations for CP and EAA appear to be above the requirements for part of the grower period and the entire finisher I and II periods.

### Effect of diet, gender and BW on the amino acid profile in the empty body

The AA requirements of growing pigs depend on the AA composition of tissue protein (Bikker *et al*., 1994) which is, when multiplied with relative deposition, equivalent to the net requirements. For the sake of simplicity, the AA-to-lysine ratio of tissue protein has been considered to be constant over the whole growth period. However, the AA-to-lysine ratios of the EB protein were highly variable among individual pigs, but they were unaffected by EBW, diet and gender, except for three AA studied. These were the phenylalanine-to-lysine and threonine-to-lysine ratios, which decreased from 52.8 (20 kg BW) to 51.1 (140 kg BW) and 55.8 (20 kg BW) to 53.6 (140 kg BW), respectively (Supplementary Figure S1). By contrast, the histidine-to-lysine ratio increased from 40 (20 kg BW) to 47 (140 kg). Out of the five fractions which constitute the EB, the carcass had the lowest phenylalanine-to-lysine ratio (Supplementary Table S4). Thus, the increasing relative importance of this fraction with respect to increasing EBW (allometric *b* coefficients for carcass fraction was >1, Supplementary Table S1) might explain the decrease of the phenylalanine-to-lysine ratio. Concomitantly, the decrease in the threonine-to-lysine ratio, which is greatest in organs and the intestine, could be explained by the decrease in the relative weight of this fraction with increasing EBW (allometric *b* coefficients for organs and the intestine fraction was <1, Supplementary Table S1). The decreasing relative weight of the organs and intestines fraction, where the lowest histidine-to-lysine ratios were determined, can explain the increase of this ratio in the EB. Previous studies have already questioned the use of constant AA profiles in tissue proteins for the elaboration of feeding recommendations (Conde-Aguilera *et al*., 2014). Although it appears that the lysine-to-protein ratio is constant, differences in three AA-to-lysine ratios were observed with increasing EBW. Further experimental data needs to be available to confirm or disprove this observation.

Phenylalanine-, leucine- and isoleucine-to-lysine ratios in the EB of the C-pigs were similar to those reported by NRC (2012). Comparing the EAA-to-lysine ratios published by NRC (2012), the present data are in agreement with respect to phenylalanine-, leucine- and isoleucine-to-lysine ratios in the EB of C-pigs, whereas valine-to-lysine and threonine-to-lysine ratios were 3 (69 *v*. 66) and 2 (55 *v*. 53) units greater in the present study. Although, from all five EB fractions, the carcass had the lowest valine-to-lysine ratio, the ratio was already >66 in C-CA and C-FE. Therefore, the remaining fractions must account for the greater ratio when compared with NRC (2012). The difference in the threonine-to-lysine ratio of the EB found in the present study compared with values reported by NRC (2012) can be explained by the greater ratios determined in increasing order in the carcass (55), organs and intestines (61) and hairs and hooves (103). Markedly lower AA-to-lysine ratios between the present study and the NRC (2012) data were found for cystine (9 *v*. 14), methionine (21 *v*. 28) and tyrosine (34 *v*. 38). Although hairs and hooves accounted for the largest cystine-to-lysine ratios (154 in C-CA and C-FE), this did not compensate for the low ratios determined in the carcass (most of them were <6). Furthermore, values reported in the literature for methionine- and methionine+cystine-to-lysine ratios in the EB, range from 25 to 39 (Kyriazakis *et al*., 1993) to 35 to 60 (Kemm *et al*., 1990). Cystine is partially degraded during hydrolysis and the yield is estimated to range between 70% and 90%. Therefore, results involving cystine should be interpreted with caution, due to this uncertain loss during the AA analysis. This points toward a large variability for these two AA with respect to lysine amount. The greatest tyrosine-to-lysine ratios were found in the hair and hooves (81) fraction which accounts for the smaller weight proportion in the EBW and therefore could not compensate for the low values (<36) found in the carcass and blood fractions. Overall, part of the differences between the proposed values by NRC (2012) and the present study for the cystine-, methionine- and tyrosine-to-lysine ratios might be explained by the greater relative proportion of the carcass fraction in modern pigs compared with the pigs used in studies decades ago.

## Conclusion

The lower relative protein and AA deposition rate together with the greater relative fat deposition rate in the EB of LP-EM compared with C-EM showed that the 20% dietary restriction of CP, lysine, methionine, tryptophan and threonine points toward a protein deficiency. As this was not true for FE and CA, one could conclude that protein and AA requirements of these genders compared with EM differ to a greater extent than currently assumed. For EM, it remains to be clarified to which extent the LP effect can be explained by a general AA deficiency or by imbalances in AA-to-lysine ratios or both. In the finisher I and II period, relative protein and AA deposition rates increased and that of fat decreased in CA and FE pigs fed the LP compared with the C-diets. This would point toward lower CP and AA requirements for CA and FE pigs in the finisher phase. However, it remains unclear to which extent the relative deposition rates of AA, CP and fat and the CP use efficiency in the finisher periods are affected by the low CP supply during the grower phase and thus represent a form of compensatory protein retention. The observed high variation of the AA-to-lysine ratios in the EB among pigs could not be attributed to neither the effect of diet, gender nor BW. Compared to current feeding recommendations for grower-finisher pigs (NRC, 2012; Agroscope, 2017), the present study revealed differences in some AA-to-lysine ratios. However, these differences to the current standards warrant verification in future studies. Apart from changes in the relative weight of each body fraction during the last decades (i.e. increased relative weight of the carcass fraction), the differences in the AA-to-lysine ratios could be a side effect of the genetic selection for different traits in the last decades.

## Supplementary material

For supplementary material accompanying this paper visit https://doi.org/10.1017/S1751731118000770. click here to view supplementary material
