# Supplementary material for: Dietary CP and amino acid restriction has a different impact on the dynamics of protein, amino acid and fat deposition in entire male, castrated and female pigs
Source: Animal. 2018 May 23;13(1):74–82. doi: 10.1017/S1751731118000770 (PMC6316349; doi:10.1017/S1751731118000770)
Supplement: Supplementary file 1 [file S1751731118000770sup001.docx]

***animal***

**Dietary crude protein and amino acid restriction has a different impact on the dynamics of protein, amino acid and fat deposition in entire male, castrated and female pigs.**

I. Ruiz-Ascacibar, P. Stoll, M. Kreuzer, and G. Bee

***Supplementary Table S1****. Allometric coefficients^1^ calculated for each gender (entire males [EM], castrates [CA], and female [FE] pigs) and within each dietary treatment (control [C] and low protein [LP])^2^ (C-EM, C-CA, C-FE, LP-EM, LP-CA and LP-FE) and for the three main body fractions (carcass, organs, blood).*

| Item | Diet | Gender | a | ASE^3^  of a | b | ASE^3^  of b | R^2^ |
| --- | --- | --- | --- | --- | --- | --- | --- |
| Carcass | C | EM | 0.651 | 0.0144 | 1.047 | 0.0048 | 1.000 |
|  |  | CA | 0.632 | 0.0138 | 1.058 | 0.0047 | 1.000 |
|  |  | FE | 0.672 | 0.0151 | 1.044 | 0.0048 | 1.000 |
|  | LP | EM | 0.653 | 0.0113 | 1.045 | 0.0037 | 1.000 |
|  |  | CA | 0.632 | 0.0144 | 1.058 | 0.0049 | 1.000 |
|  |  | FE | 0.665 | 0.0157 | 1.047 | 0.0051 | 1.000 |
| Organs | C | EM | 0.319 | 0.0309 | 0.808 | 0.0211 | 0.991 |
|  |  | CA | 0.370 | 0.0379 | 0.751 | 0.0224 | 0.984 |
|  |  | FE | 0.296 | 0.0285 | 0.802 | 0.0210 | 0.991 |
|  | LP | EM | 0.287 | 0.0190 | 0.838 | 0.0144 | 0.996 |
|  |  | CA | 0.372 | 0.0370 | 0.754 | 0.0217 | 0.985 |
|  |  | FE | 0.325 | 0.0333 | 0.781 | 0.0224 | 0.989 |
| Blood | C | EM | 0.106 | 0.0141 | 0.821 | 0.0289 | 0.984 |
|  |  | CA | 0.150 | 0.0317 | 0.723 | 0.0465 | 0.931 |
|  |  | FE | 0.130 | 0.0191 | 0.767 | 0.0322 | 0.977 |
|  | LP | EM | 0.118 | 0.0182 | 0.798 | 0.0337 | 0.976 |
|  |  | CA | 0.129 | 0.0287 | 0.755 | 0.0484 | 0.931 |
|  |  | FE | 0.132 | 0.0181 | 0.751 | 0.0301 | 0.978 |

The allometric regression used was Y = a × EBW^b^, where Y = predicted amount (g) of EB protein, amino acid or fat; EBW = empty body weight (kg); b = allometric coefficient; a = constant. R^2^ is based on the original and untransformed data.

^2^The C diets were formulated to meet nutrient requirement for grower finisher pigs in the grower, finisher I and finisher II periods according to the Swiss feeding recommendations for pigs; the LP diets was formulated to contain, expressed as percentage of C diets, 80% of dietary CP, lysine, methionine + cystine, threonine and tryptophan.

^3^ASE= Asymptotic standard error

Supplementary Material S1**.** Detailed description of the pre-treatment of the data of empty body (EB) protein, amino acid (AA) and fat content in order to correct for the corresponding standardized empty body weight (EBW) categories.

1. A preliminary allometric regression (Y= a×EBW^b^) was calculated for each gender (entire males, EM, castrates, CA and female FE pigs) and within each dietary treatment (control, C and low protein, LP) (C-EM, C-CA, C-FE, LP-EM, LP-CA and LP-FE) and for each EB component (protein, methionine, cystine, threonine, phenylalanine, tyrosine, valine, leucine and isoleucine and fat) (Supplementary Table S1).
2. One average, EBW was calculated per BW category from 20 to 140 kg EBW (18.3, 39.8, 56.7, 78.3, 95.9, 116.0, 133.9 kg)
3. These average EBW data were used, together with the preliminary allometric equations, to calculate EB protein, AA and fat contents for each gender and diet at each EBW category.
4. This preliminary equation was used to estimate the content of each EB component for each animal according to its own EBW.
5. The difference between the estimated EB protein, AA and fat contents for each animal according to its own EBW and the corresponding value of component content, calculated for each average EBW, was used to correct the individual protein, AA and fat contents assuming that all pigs would have had the same EBW at slaughter.

**Supplementary Table S2**. *Protein, fat, lysine (Lys), methionine (Met), cystine (Cys), threonine (Thr), phenylalanine (Phe), tyrosine (Tyr), valine (Val), leucine (Leu), isoleucine (Ile) and histidine (His) contents in g of the empty body of female (FE), castrated (CA) and entire male (EM) pigs fed the control (C) or low CP (LP) grower (20-60 kg), finisher I (60-100 kg) and finisher II (100-140 kg) diets*^1^ *and slaughtered either the day of birth and 10, 20, 40, 60, 80, 100, 120 or 140 kg body BW.*

| BW  category (kg) | Diet^1^ | Gender | BW  (kg) | EBW^2^  (kg) | Protein | Fat | Lys | Met | Cys | Thr | Phe | Tyr | Val | Leu | Ile | His |
| --- | --- | --- | --- | --- | --- | --- | --- | --- | --- | --- | --- | --- | --- | --- | --- | --- |
| new born |  | EM | 1.5 | 1.4 | 158.4 | 37.4 | 9.0 | 2.2 | 1.0 | 5.6 | 5.1 | 3.0 | 7.0 | 9.7 | 4.9 | 3.7 |
|  |  | FE | 1.3 | 1.3 | 156.9 | 25.6 | 8.3 | 2.0 | 0.9 | 5.0 | 4.7 | 2.7 | 6.4 | 8.9 | 4.5 | 3.3 |
|  |  |  |  |  |  |  |  |  |  |  |  |  |  |  |  |  |
| 10 | Starter diet | EM | 9.5 | 8.9 | 1 357.0 | 1 055.0 | 81.8 | 21.3 | 5.9 | 46.8 | 45.0 | 28.5 | 59.8 | 88.7 | 43.5 | 34.1 |
|  |  | CA | 9.9 | 9.0 | 1 360.5 | 961.3 | 65.1 | 16.6 | 5.8 | 36.6 | 37.3 | 23.1 | 49.2 | 70.4 | 32.4 | 27.7 |
|  |  | FE | 9.6 | 8.7 | 1 259.8 | 846.1 | 77.3 | 20.0 | 4.9 | 43.8 | 42.1 | 26.1 | 55.5 | 81.5 | 40.6 | 31.7 |
|  |  |  |  |  |  |  |  |  |  |  |  |  |  |  |  |  |
| 20 | Starter diet | EM | 19.6 | 18.3 | 2 846.4 | 1 583.6 | 196.3 | 41.8 | 19.7 | 109.5 | 102.9 | 63.6 | 139.3 | 200.1 | 98.6 | 77.9 |
|  |  | CA | 21.2 | 19.9 | 3 187.8 | 2 127.7 | 213.2 | 45.2 | 21.7 | 121.1 | 114.7 | 68.2 | 155.0 | 220.7 | 107.4 | 86.4 |
|  |  | FE | 21.6 | 20.2 | 3 367.2 | 1 988.4 | 225.1 | 44.4 | 21.3 | 122.6 | 118.1 | 73.4 | 158.4 | 227.2 | 112.4 | 90.0 |
|  |  |  |  |  |  |  |  |  |  |  |  |  |  |  |  |  |
| 40 | C | EM | 41.7 | 39.8 | 6 715.0 | 4 698.1 | 457.9 | 116.0 | 49.5 | 255.0 | 241.7 | 154.8 | 324.9 | 466.1 | 238.2 | 191.8 |
|  |  | CA | 40.4 | 38.5 | 6 201.4 | 5 540.7 | 448.0 | 88.5 | 46.6 | 255.6 | 236.2 | 149.4 | 317.0 | 445.4 | 222.4 | 184.8 |
|  |  | FE | 40.9 | 39.1 | 6 572.7 | 5 227.4 | 462.4 | 86.3 | 40.5 | 251.9 | 234.9 | 145.8 | 315.1 | 452.6 | 230.9 | 206.9 |
|  | LP | EM | 38.6 | 36.1 | 5 599.2 | 5 180.3 | 328.6 | 76.4 | 34.0 | 184.1 | 182.6 | 114.6 | 235.5 | 337.6 | 161.8 | 143.3 |
|  |  | CA | 40.7 | 37.8 | 5 945.7 | 5 829.6 | 369.2 | 85.8 | 32.4 | 203.9 | 198.9 | 123.6 | 257.5 | 368.2 | 182.5 | 162.9 |
|  |  | FE | 39.7 | 37.0 | 5 874.6 | 5 372.8 | 416.6 | 82.8 | 32.2 | 225.2 | 212.7 | 145.7 | 286.2 | 412.2 | 214.2 | 174.1 |
|  |  |  |  |  |  |  |  |  |  |  |  |  |  |  |  |  |
| 60 | C | EM | 59.5 | 56.7 | 9 728.8 | 6 605.1 | 684.8 | 147.7 | 68.5 | 371.1 | 368.0 | 217.1 | 478.9 | 685.1 | 339.0 | 280.8 |
|  |  | CA | 63.1 | 60.2 | 10 226.6 | 9 735.6 | 723.8 | 171.0 | 71.2 | 392.7 | 379.8 | 250.9 | 499.5 | 718.1 | 367.5 | 314.6 |
|  |  | FE | 64.2 | 61.3 | 10 226.7 | 8 988.3 | 735.9 | 164.9 | 58.4 | 399.2 | 374.8 | 240.6 | 499.8 | 731.9 | 369.3 | 323.2 |
|  | LP | EM | 63.0 | 59.1 | 9 442.6 | 9 448.1 | 603.1 | 121.7 | 47.5 | 369.1 | 330.1 | 221.2 | 443.9 | 635.7 | 321.9 | 296.5 |
|  |  | CA | 60.4 | 57.7 | 8 901.3 | 11 304.6 | 610.3 | 137.4 | 54.1 | 336.6 | 317.8 | 211.6 | 421.9 | 612.9 | 306.1 | 272.5 |
|  |  | FE | 61.6 | 58.5 | 9 433.2 | 10 596.6 | 616.9 | 144.8 | 58.0 | 343.3 | 339.8 | 212.4 | 444.5 | 626.5 | 312.9 | 279.8 |
|  |  |  |  |  |  |  |  |  |  |  |  |  |  |  |  |  |
| 80 | C | EM | 82.3 | 78.3 | 13 821.9 | 11 665.4 | 973.1 | 182.4 | 84.7 | 545.6 | 492.1 | 330.7 | 669.3 | 964.0 | 493.7 | 454.8 |
|  |  | CA | 80.5 | 77.3 | 13 072.4 | 14 139.6 | 872.0 | 144.7 | 67.6 | 497.7 | 443.4 | 310.2 | 606.0 | 885.1 | 459.8 | 434.5 |
|  |  | FE | 79.4 | 76.0 | 12 905.5 | 13 310.6 | 796.2 | 189.1 | 76.9 | 434.4 | 420.0 | 272.6 | 562.0 | 802.7 | 406.0 | 368.3 |
|  | LP | EM | 84.1 | 78.8 | 12 803.0 | 13 609.6 | 882.5 | 171.8 | 70.9 | 518.2 | 448.5 | 293.9 | 612.3 | 901.7 | 464.4 | 427.8 |
|  |  | CA | 81.0 | 77.3 | 12 121.6 | 16 511.2 | 881.8 | 184.8 | 73.8 | 478.0 | 438.3 | 285.5 | 597.9 | 864.8 | 440.7 | 408.7 |
|  |  | FE | 79.3 | 75.3 | 12 153.5 | 15 100.8 | 858.6 | 191.8 | 75.8 | 479.6 | 453.7 | 301.5 | 605.2 | 869.5 | 436.2 | 390.7 |
|  |  |  |  |  |  |  |  |  |  |  |  |  |  |  |  |  |
| 100 | C | EM | 100.4 | 95.9 | 16 713.7 | 16 074.8 | 1 222.1 | 250.3 | 106.9 | 683.6 | 625.6 | 416.1 | 844.0 | 1 210.2 | 623.9 | 561.7 |
|  |  | CA | 102.2 | 97.7 | 16 584.5 | 20 284.2 | 1 047.1 | 214.7 | 114.1 | 605.4 | 556.6 | 350.1 | 748.5 | 1 091.5 | 555.2 | 518.2 |
|  |  | FE | 100.8 | 96.5 | 16 348.0 | 17 392.3 | 1 154.6 | 240.9 | 97.8 | 618.6 | 581.4 | 393.9 | 790.2 | 1 118.8 | 587.4 | 533.4 |
|  | LP | EM | 99.9 | 95.5 | 15 456.3 | 18 982.9 | 974.1 | 206.4 | 76.5 | 531.8 | 503.6 | 315.2 | 681.0 | 992.3 | 485.3 | 524.0 |
|  |  | CA | 100.9 | 97.1 | 15 019.8 | 24 703.0 | 1 129.1 | 272.0 | 97.4 | 627.1 | 579.2 | 396.5 | 769.9 | 1 141.0 | 581.6 | 541.1 |
|  |  | FE | 102.4 | 97.2 | 16 027.6 | 21 058.2 | 1 000.1 | 173.5 | 81.9 | 580.2 | 518.3 | 331.8 | 691.3 | 1 029.7 | 518.7 | 511.5 |
|  |  |  |  |  |  |  |  |  |  |  |  |  |  |  |  |  |
| 120 | C | EM | 119.8 | 116.0 | 19 812.4 | 21 080.9 | 1 267.0 | 229.7 | 109.1 | 708.8 | 643.8 | 441.5 | 876.4 | 1 276.5 | 652.4 | 593.2 |
|  |  | CA | 117.9 | 114.3 | 18 709.8 | 27 410.2 | 1 195.2 | 272.1 | 113.2 | 673.2 | 628.6 | 427.7 | 832.1 | 1 255.5 | 624.2 | 513.1 |
|  |  | FE | 118.0 | 114.2 | 18 987.2 | 25 147.7 | 1 288.4 | 287.3 | 101.7 | 712.8 | 661.4 | 443.8 | 868.8 | 1 289.4 | 669.1 | 673.6 |
|  | LP | EM | 120.4 | 115.8 | 19 326.2 | 23 166.7 | 1 263.0 | 246.8 | 105.4 | 670.3 | 627.2 | 415.2 | 848.9 | 1 249.4 | 639.8 | 557.7 |
|  |  | CA | 120.9 | 116.2 | 19 398.1 | 30 398.1 | 1 234.5 | 278.7 | 112.0 | 642.2 | 622.1 | 429.5 | 838.8 | 1 226.0 | 628.8 | 554.6 |
|  |  | FE | 122.4 | 116.6 | 19 172.8 | 28 645.1 | 1 231.9 | 279.5 | 109.2 | 625.0 | 607.6 | 427.3 | 810.8 | 1 235.2 | 639.0 | 548.9 |
|  |  |  |  |  |  |  |  |  |  |  |  |  |  |  |  |  |
| 140 | C | EM | 138.3 | 133.9 | 23 333.6 | 24 514.4 | 1 674.8 | 363.3 | 140.8 | 921.4 | 864.9 | 587.4 | 1 173.1 | 1 678.9 | 874.2 | 743.1 |
|  |  | CA | 139.6 | 136.2 | 21 455.1 | 38  49.5 | 1 363.2 | 270.5 | 120.0 | 716.7 | 715.8 | 455.4 | 932.5 | 1 381.9 | 688.0 | 679.2 |
|  |  | FE | 141.2 | 134.9 | 21 434.8 | 35 018.5 | 1 296.6 | 276.5 | 121.9 | 715.2 | 654.0 | 442.0 | 875.4 | 1 343.9 | 685.0 | 647.6 |
|  | LP | EM | 142.9 | 137.5 | 21 864.7 | 33 570.3 | 1 356.7 | 255.2 | 123.8 | 730.1 | 684.2 | 460.3 | 909.6 | 1 353.3 | 689.9 | 626.4 |
|  |  | CA | 143.1 | 138.0 | 20 836.0 | 40 299.7 | 1 388.8 | 299.9 | 141.1 | 713.3 | 694.0 | 467.5 | 930.5 | 1 385.1 | 711.9 | 631.1 |
|  |  | FE | 141.0 | 135.7 | 21 917.3 | 34 465.8 | 1 535.3 | 347.6 | 165.9 | 827.1 | 783.6 | 533.6 | 1 081.4 | 1 566.1 | 812.3 | 702.1 |

^1^ Control diets (C) were formulated to meet nutrient requirements according to the standard Swiss feeding recommendations for grower finisher pigs in the respective growth periods; low protein diets (LP) were formulated to contain, expressed as percentage of the control diets, 80% of dietary CP, lysine, methionine + cystine, threonine and tryptophan.

^2^ EBW = Empty body weight

*Supplementary Table S3. Preliminary allometric coefficients^1^ calculated for each gender (entire males [EM], castrates [CA], and female [FE] pigs) and within each dietary treatment (control [C] and low protein [LP])^2^ (C-EM, C-CA, C-FE, LP-EM, LP-CA and LP-FE) and for each EB component (protein, methionine, cystine, threonine, phenylalanine, tyrosine, valine, leucine and isoleucine and fat).*

| Item | Diet^2^ | Gender | a | ASE^3^ of a | b | ASE^3^ of b | R^2^ |
| --- | --- | --- | --- | --- | --- | --- | --- |
| Protein | C | EM | 153.4 | 17.66 | 1.026 | 0.0248 | 0.993 |
|  |  | CA | 194.8 | 20.95 | 0.962 | 0.0232 | 0.992 |
|  |  | FE | 195.1 | 24.72 | 0.963 | 0.0274 | 0.990 |
|  | LP | EM | 153.1 | 10.87 | 1.012 | 0.0153 | 0.997 |
|  |  | CA | 163.4 | 21.58 | 0.991 | 0.0284 | 0.988 |
|  |  | FE | 160.8 | 11.08 | 1.003 | 0.0148 | 0.997 |
| Fat | C | EM | 25.3 | 9.22 | 1.409 | 0.0776 | 0.967 |
|  |  | CA | 8.8 | 3.24 | 1.703 | 0.0778 | 0.977 |
|  |  | FE | 7.9 | 3.33 | 1.705 | 0.0885 | 0.970 |
|  | LP | EM | 19.9 | 5.37 | 1.502 | 0.0570 | 0.984 |
|  |  | CA | 27.1 | 6.59 | 1.481 | 0.0513 | 0.986 |
|  |  | FE | 30.0 | 5.14 | 1.437 | 0.0363 | 0.993 |
| Amino acids |  |  |  |  |  |  |  |
| Lysine | C | EM | 10.7 | 3.55 | 1.026 | 0.0715 | 0.944 |
|  |  | CA | 17.3 | 5.38 | 0.893 | 0.0674 | 0.921 |
|  |  | FE | 17.7 | 5.54 | 0.893 | 0.0680 | 0.931 |
|  | LP | EM | 10.9 | 2.64 | 0.990 | 0.0522 | 0.967 |
|  |  | CA | 12.8 | 3.49 | 0.960 | 0.0585 | 0.948 |
|  |  | FE | 10.0 | 3.17 | 1.018 | 0.0678 | 0.946 |
| Methionine | C | EM | 2.6 | 1.95 | 0.985 | 0.1601 | 0.752 |
|  |  | CA | 3.9 | 2.51 | 0.874 | 0.1401 | 0.716 |
|  |  | FE | 3.7 | 1.42 | 0.903 | 0.0842 | 0.901 |
|  | LP | EM | 3.2 | 1.30 | 0.904 | 0.0883 | 0.891 |
|  |  | CA | 3.0 | 1.30 | 0.948 | 0.0928 | 0.876 |
|  |  | FE | 1.5 | 0.82 | 1.092 | 0.1134 | 0.878 |
| Cystine | C | EM | 1.7 | 1.00 | 0.895 | 0.1272 | 0.798 |
|  |  | CA | 2.1 | 1.11 | 0.840 | 0.1168 | 0.767 |
|  |  | FE | 1.5 | 0.51 | 0.898 | 0.0736 | 0.921 |
|  | LP | EM | 0.9 | 0.38 | 1.004 | 0.0955 | 0.896 |
|  |  | CA | 0.7 | 0.37 | 1.066 | 0.1091 | 0.866 |
|  |  | FE | 0.4 | 0.22 | 1.224 | 0.1313 | 0.873 |
| Methionine+cystine | C | EM | 4.3 | 2.82 | 0.955 | 0.1416 | 0.785 |
|  |  | CA | 5.9 | 3.34 | 0.863 | 0.1221 | 0.763 |
|  |  | FE | 5.2 | 1.64 | 0.902 | 0.0688 | 0.932 |
|  | LP | EM | 4.0 | 1.36 | 0.931 | 0.0727 | 0.928 |
|  |  | CA | 3.7 | 1.53 | 0.978 | 0.0882 | 0.893 |
|  |  | FE | 1.9 | 0.86 | 1.128 | 0.0984 | 0.911 |
| Threonine | C | EM | 6.0 | 1.74 | 1.023 | 0.0624 | 0.957 |
|  |  | CA | 11.2 | 3.21 | 0.860 | 0.0625 | 0.926 |
|  |  | FE | 9.3 | 3.44 | 0.902 | 0.0805 | 0.908 |
|  | LP | EM | 8.2 | 2.36 | 0.921 | 0.0622 | 0.946 |
|  |  | CA | 9.0 | 2.77 | 0.900 | 0.0665 | 0.924 |
|  |  | FE | 6.6 | 2.09 | 0.975 | 0.0681 | 0.940 |
| Phenylalanine | C | EM | 6.0 | 1.76 | 1.008 | 0.0636 | 0.954 |
|  |  | CA | 9.0 | 2.79 | 0.895 | 0.0670 | 0.921 |
|  |  | FE | 9.8 | 2.82 | 0.874 | 0.0624 | 0.939 |
|  | LP | EM | 7.1 | 1.81 | 0.936 | 0.0551 | 0.958 |
|  |  | CA | 7.6 | 2.03 | 0.927 | 0.0578 | 0.944 |
|  |  | FE | 6.3 | 1.95 | 0.973 | 0.0666 | 0.942 |
| Tyrosine | C | EM | 2.8 | 1.17 | 1.085 | 0.0899 | 0.924 |
|  |  | CA | 5.9 | 2.35 | 0.894 | 0.0862 | 0.878 |
|  |  | FE | 5.2 | 1.82 | 0.924 | 0.0758 | 0.922 |
|  | LP | EM | 4.1 | 1.09 | 0.965 | 0.0577 | 0.957 |
|  |  | CA | 4.1 | 1.27 | 0.974 | 0.0665 | 0.937 |
|  |  | FE | 3.2 | 0.89 | 1.033 | 0.0593 | 0.959 |
| Valine | C | EM | 7.5 | 2.29 | 1.023 | 0.0654 | 0.952 |
|  |  | CA | 13.2 | 3.92 | 0.874 | 0.0645 | 0.923 |
|  |  | FE | 13.4 | 3.85 | 0.869 | 0.0624 | 0.939 |
|  | LP | EM | 9.7 | 2.54 | 0.933 | 0.0567 | 0.956 |
|  |  | CA | 9.9 | 2.75 | 0.932 | 0.0598 | 0.941 |
|  |  | FE | 7.5 | 2.40 | 1.002 | 0.0692 | 0.941 |
| Leucine | C | EM | 10.7 | 3.32 | 1.025 | 0.0668 | 0.951 |
|  |  | CA | 16.6 | 4.49 | 0.908 | 0.0584 | 0.941 |
|  |  | FE | 16.2 | 4.66 | 0.913 | 0.0624 | 0.944 |
|  | LP | EM | 12.7 | 3.28 | 0.958 | 0.0558 | 0.959 |
|  |  | CA | 13.3 | 3.43 | 0.953 | 0.0558 | 0.951 |
|  |  | FE | 9.9 | 2.96 | 1.025 | 0.0646 | 0.951 |
| Isoleucine | C | EM | 4.8 | 1.95 | 1.055 | 0.0871 | 0.924 |
|  |  | CA | 8.9 | 3.22 | 0.895 | 0.0787 | 0.896 |
|  |  | FE | 7.8 | 2.80 | 0.928 | 0.0782 | 0.917 |
|  | LP | EM | 5.9 | 1.68 | 0.977 | 0.0615 | 0.953 |
|  |  | CA | 6.1 | 1.71 | 0.975 | 0.0606 | 0.947 |
|  |  | FE | 4.4 | 1.40 | 1.055 | 0.0682 | 0.949 |
| Histidine | C | EM | 3.9 | 1.55 | 1.074 | 0.0864 | 0.928 |
|  |  | CA | 5.7 | 3.26 | 0.971 | 0.1234 | 0.812 |
|  |  | FE | 5.1 | 2.35 | 1.007 | 0.0996 | 0.891 |
|  | LP | EM | 5.6 | 1.97 | 0.973 | 0.0764 | 0.930 |
|  |  | CA | 5.5 | 2.07 | 0.975 | 0.0815 | 0.909 |
|  |  | FE | 4.0 | 1.51 | 1.051 | 0.0820 | 0.928 |

The allometric regression used was Y = a × EBW^b^, where Y = predicted amount (g) of EB protein, amino acid or fat; EBW = empty body weight (kg); b = allometric coefficient; a = constant. R^2^ is based on the original and untransformed data.

^2^ The C diets were formulated to meet nutrient requirements for grower finisher pigs in the grower, finisher I and finisher II periods according to the Swiss feeding recommendations for pigs; the LP diets were formulated to contain, expressed as percentage of C diets, 80% of dietary CP, lysine, methionine + cystine, threonine and tryptophan.

^3^ ASE= Asymptotic standard error

**
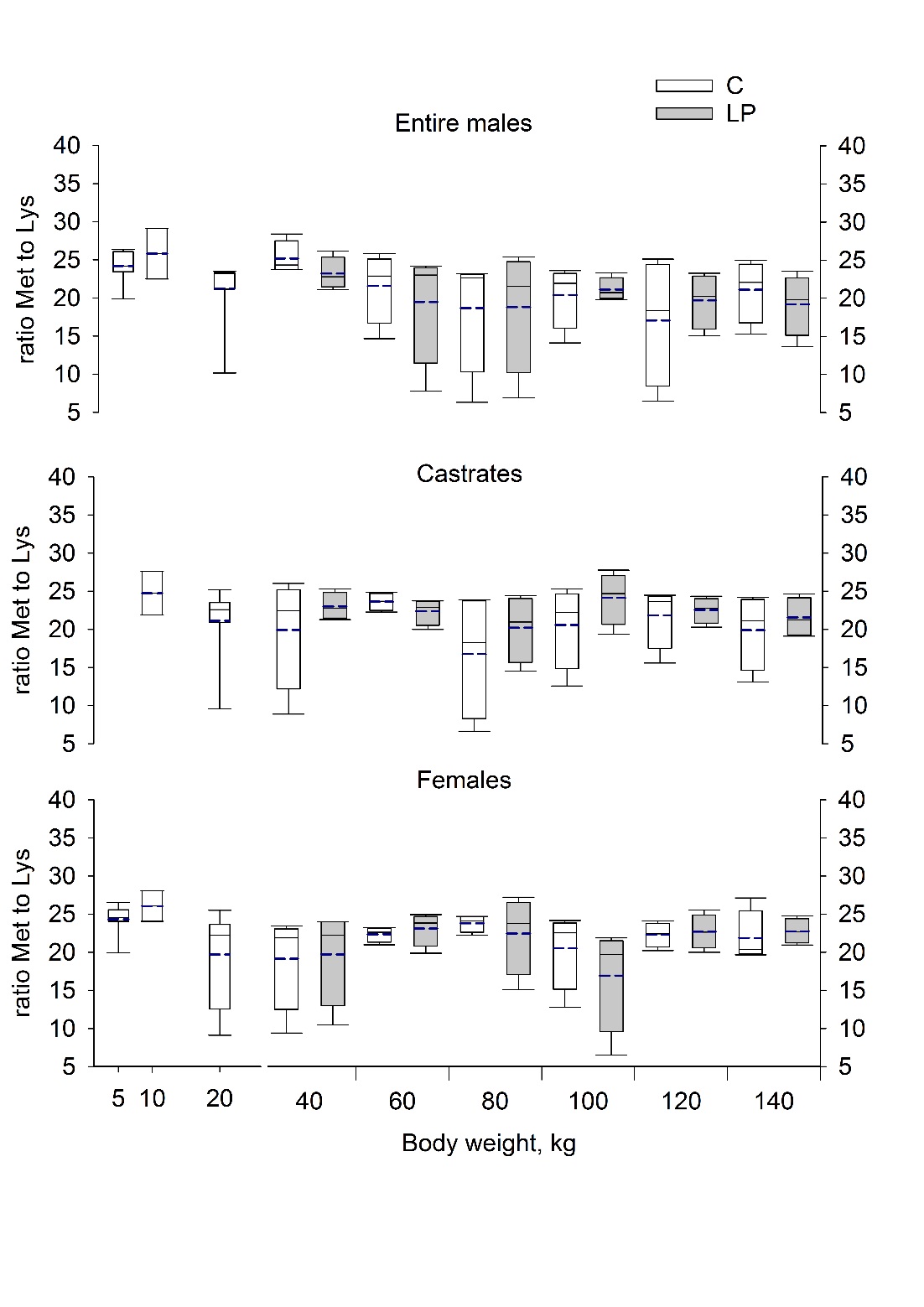

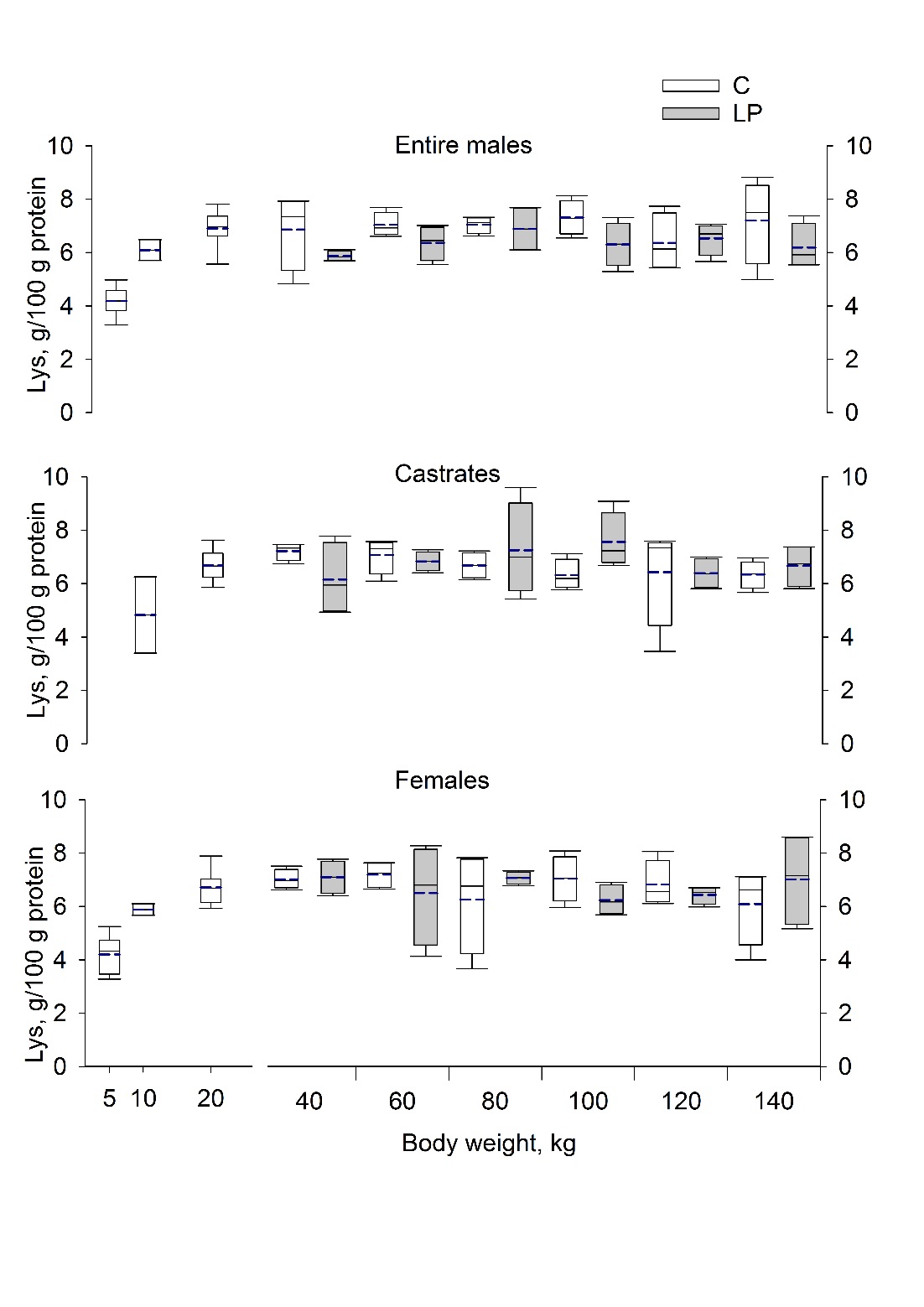
**


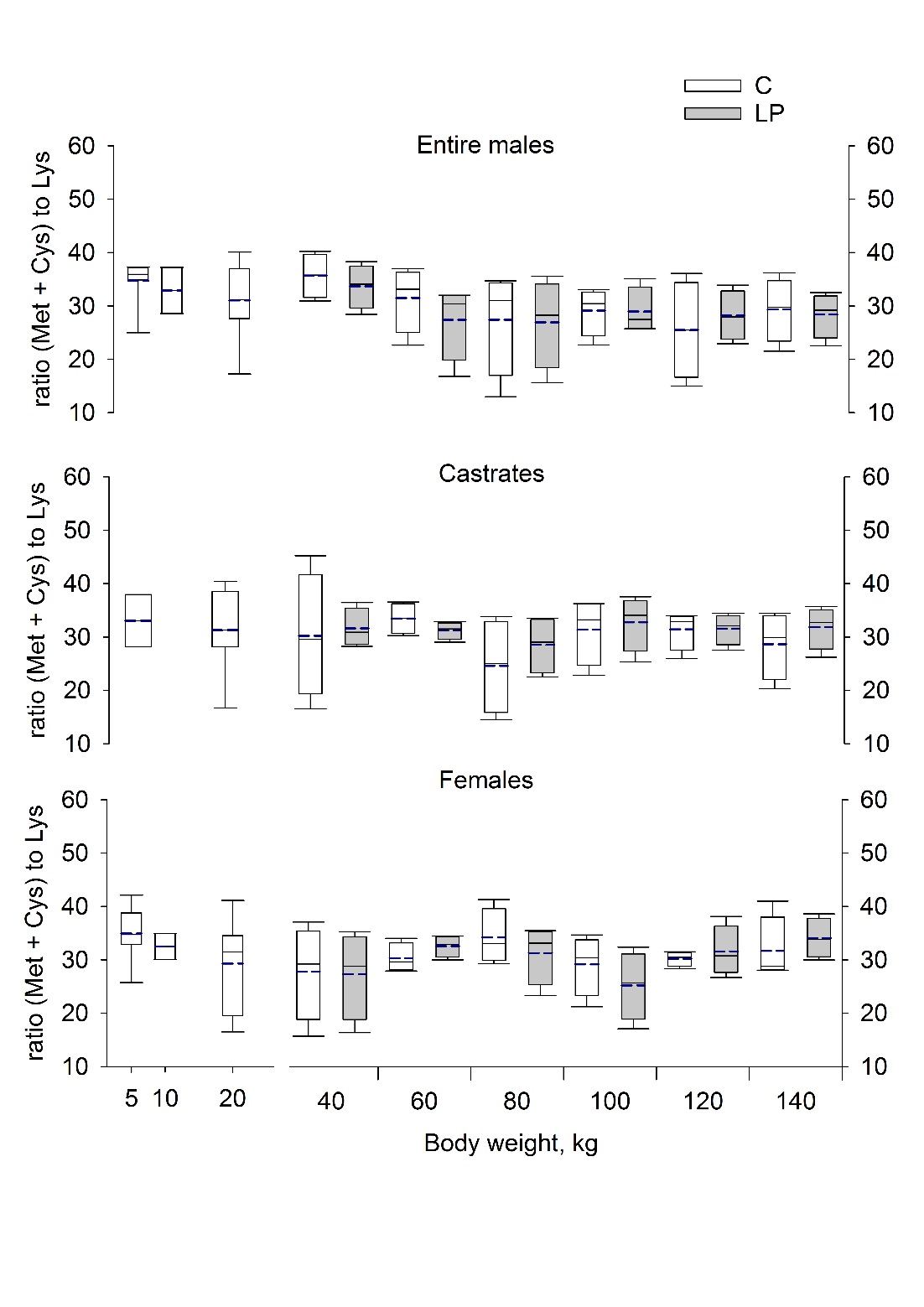

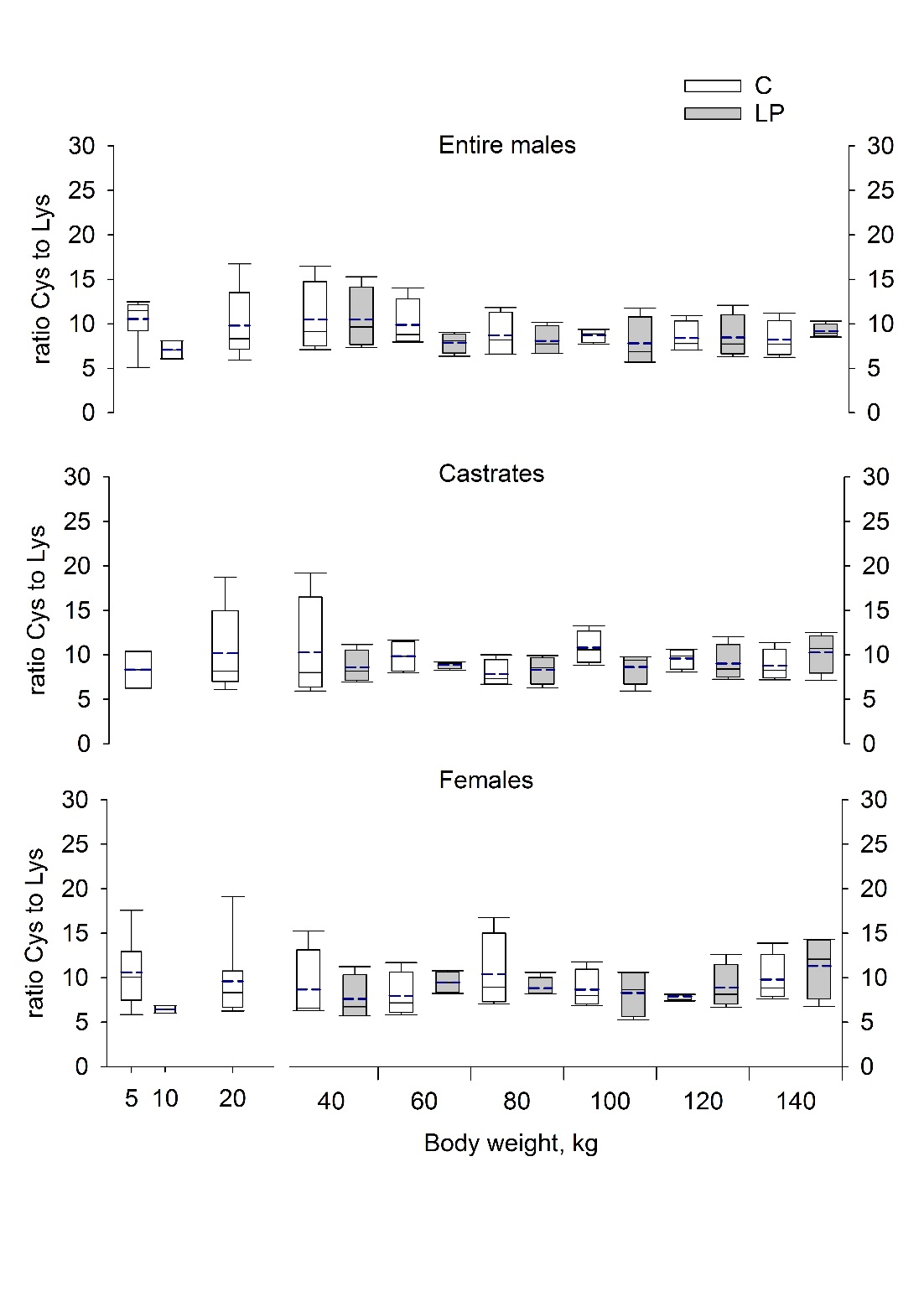


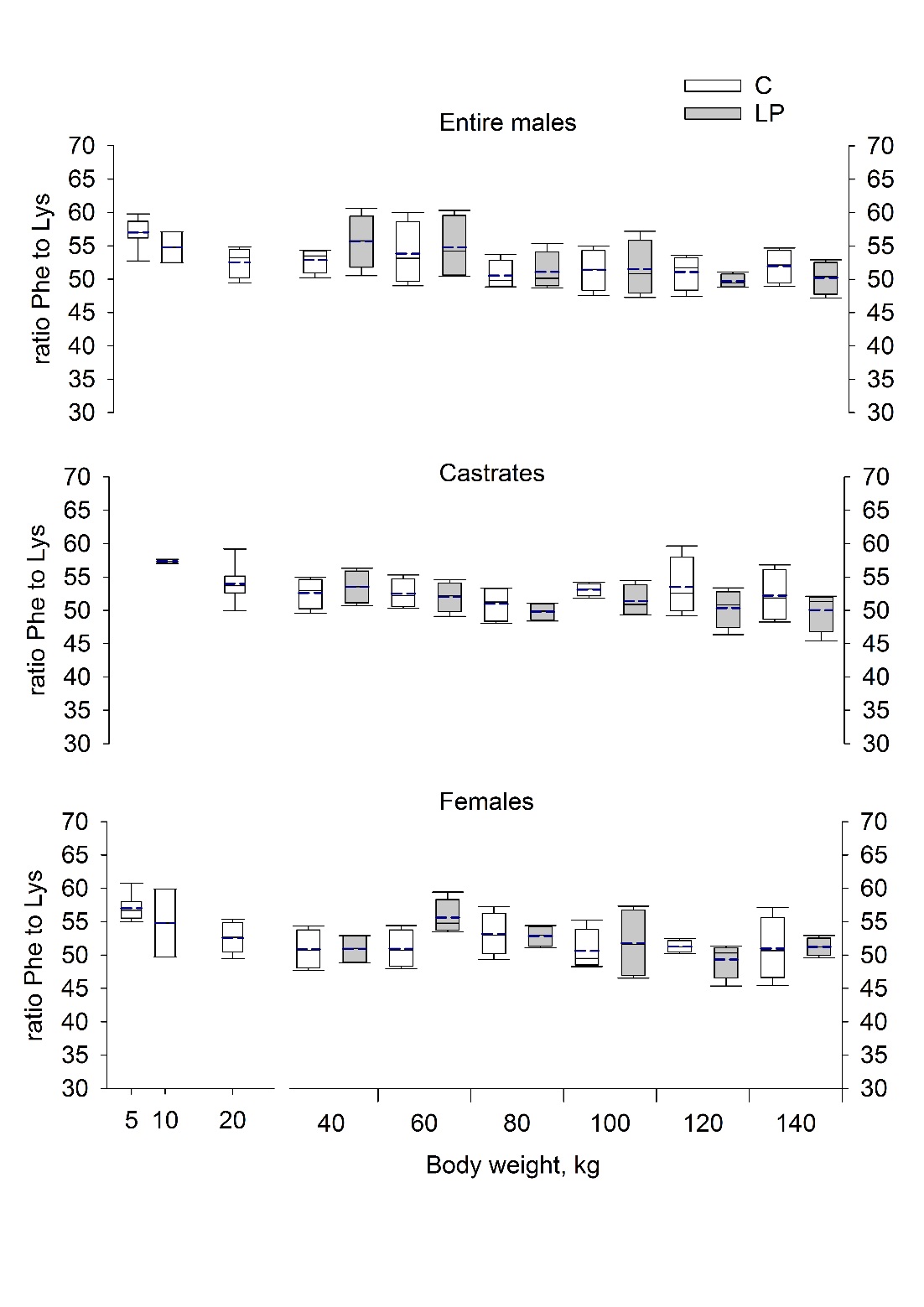

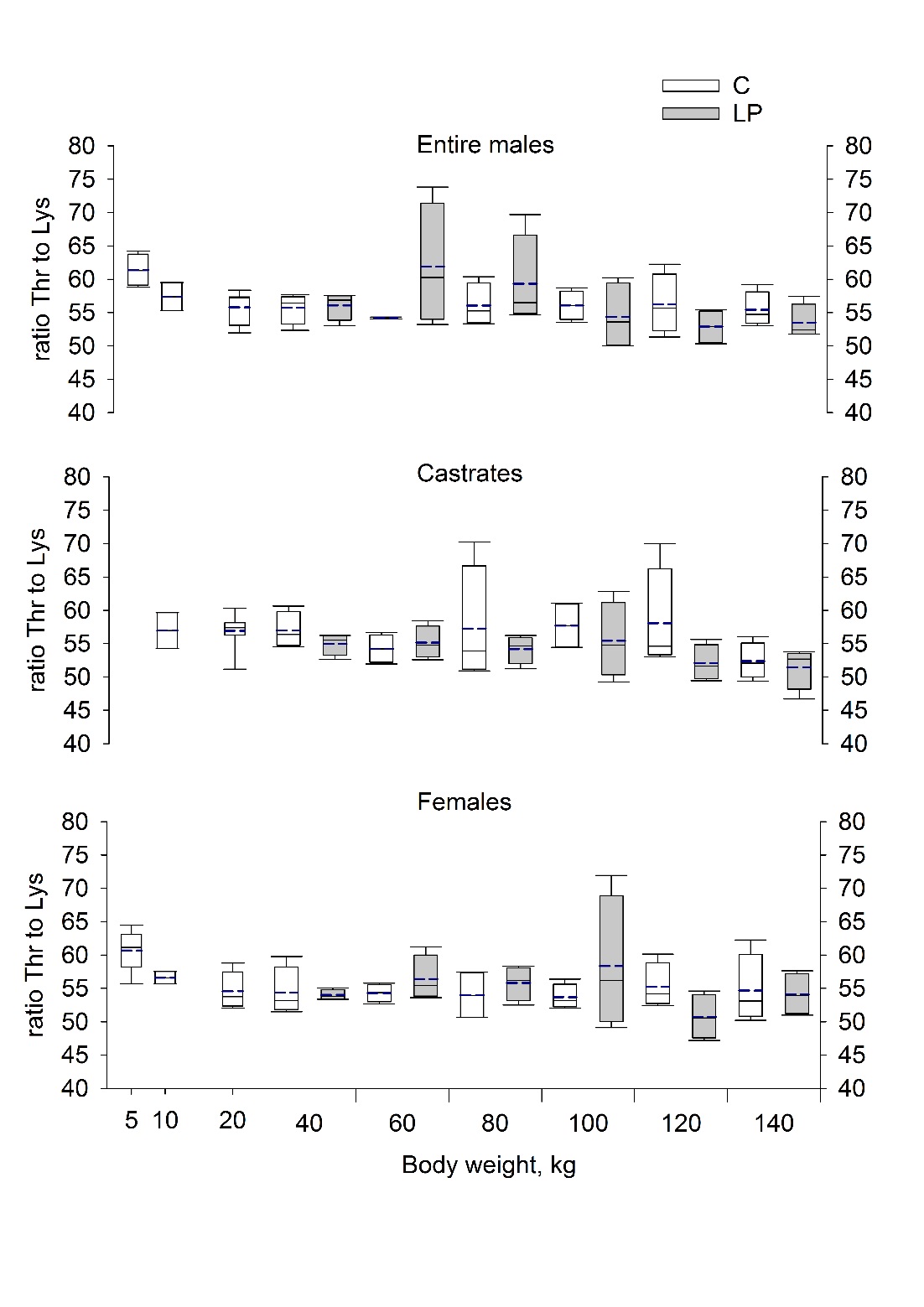


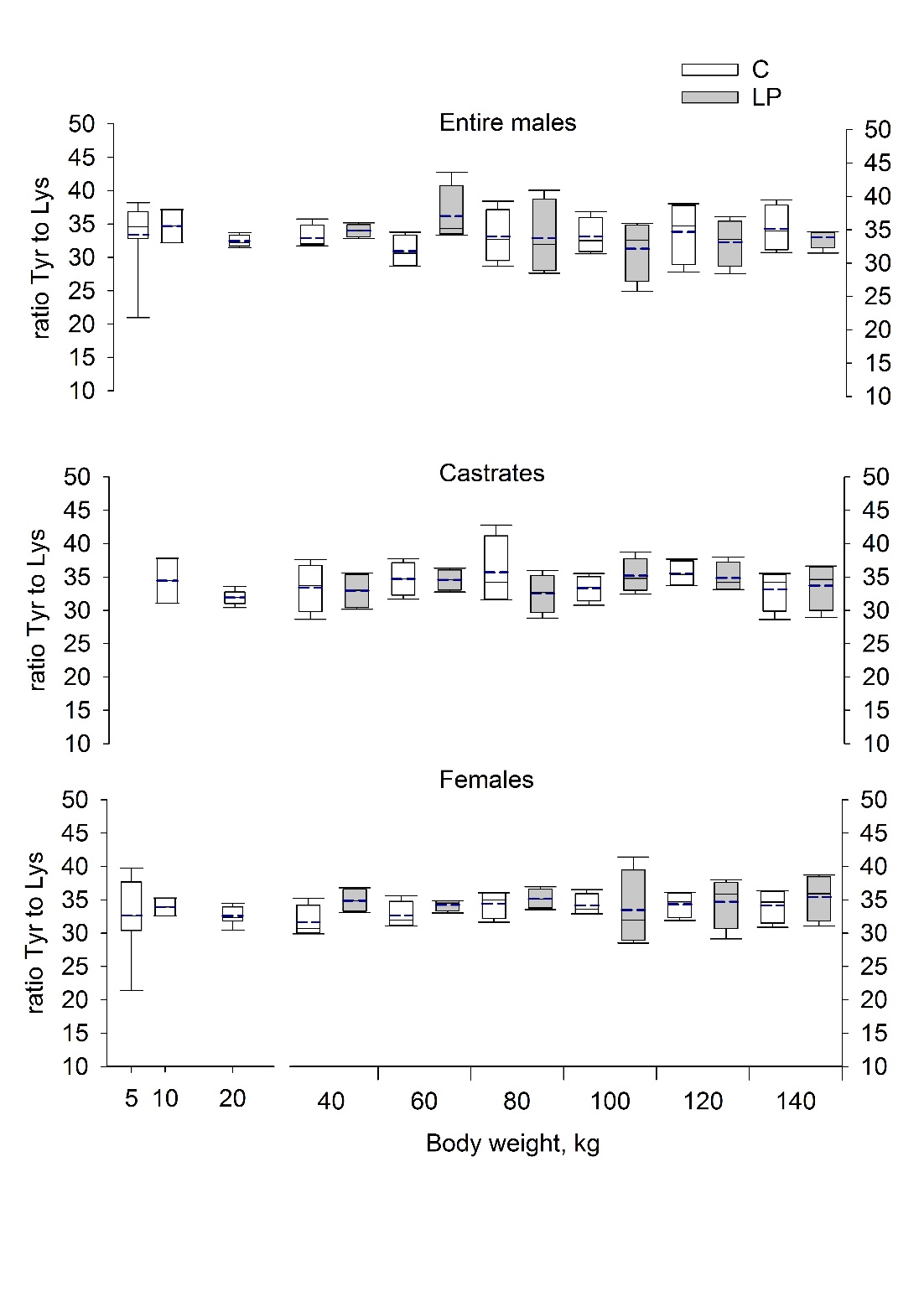

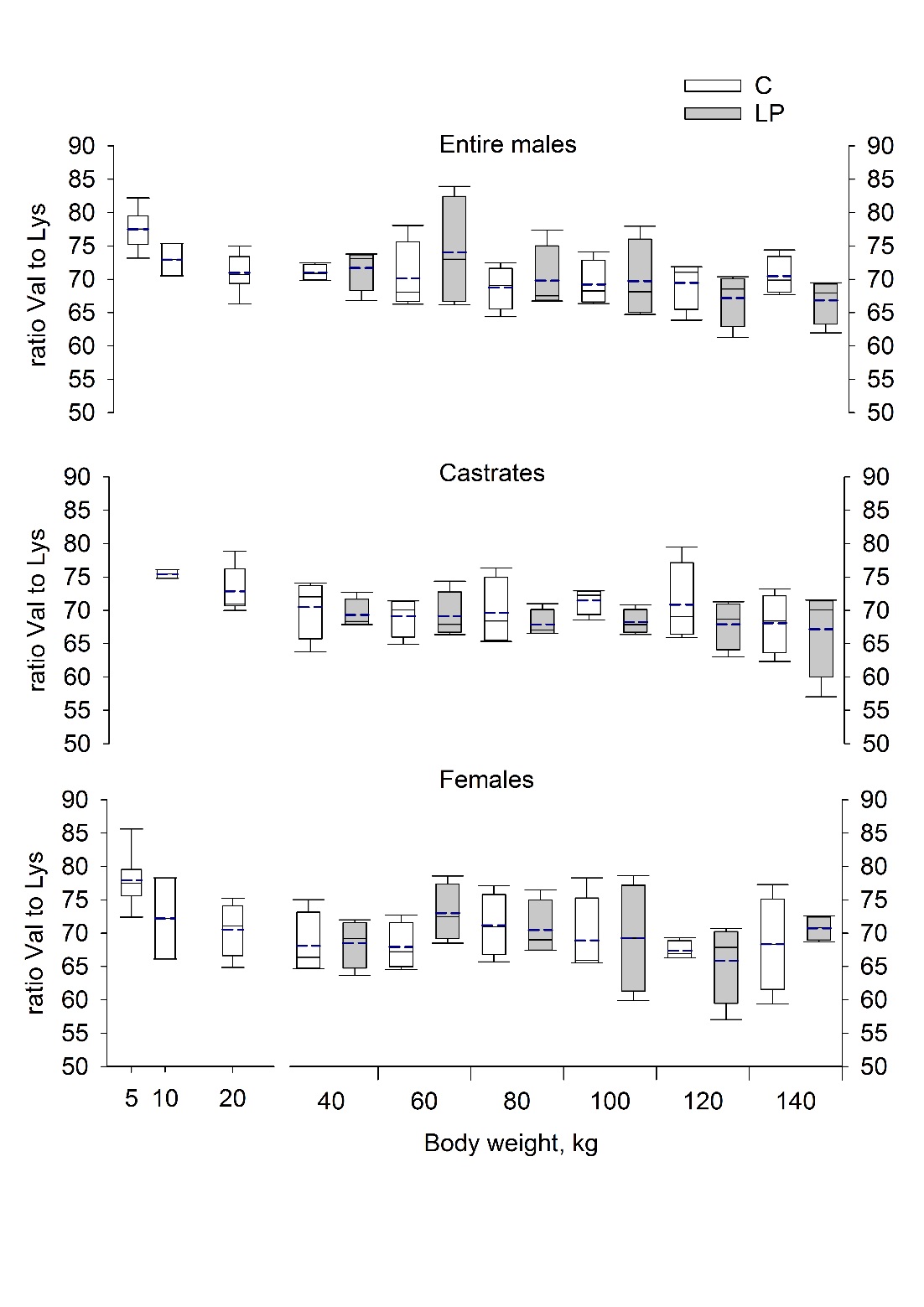


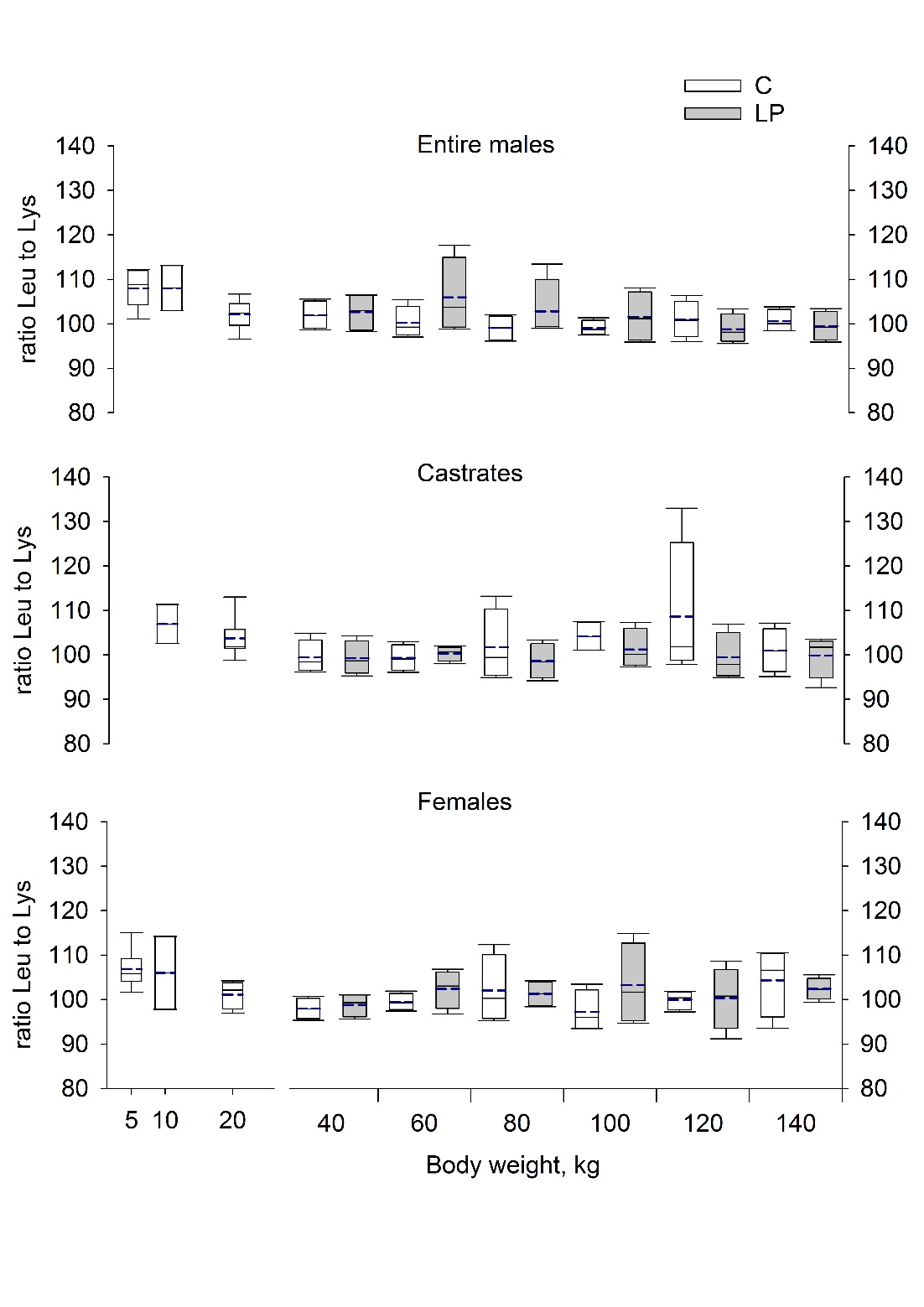

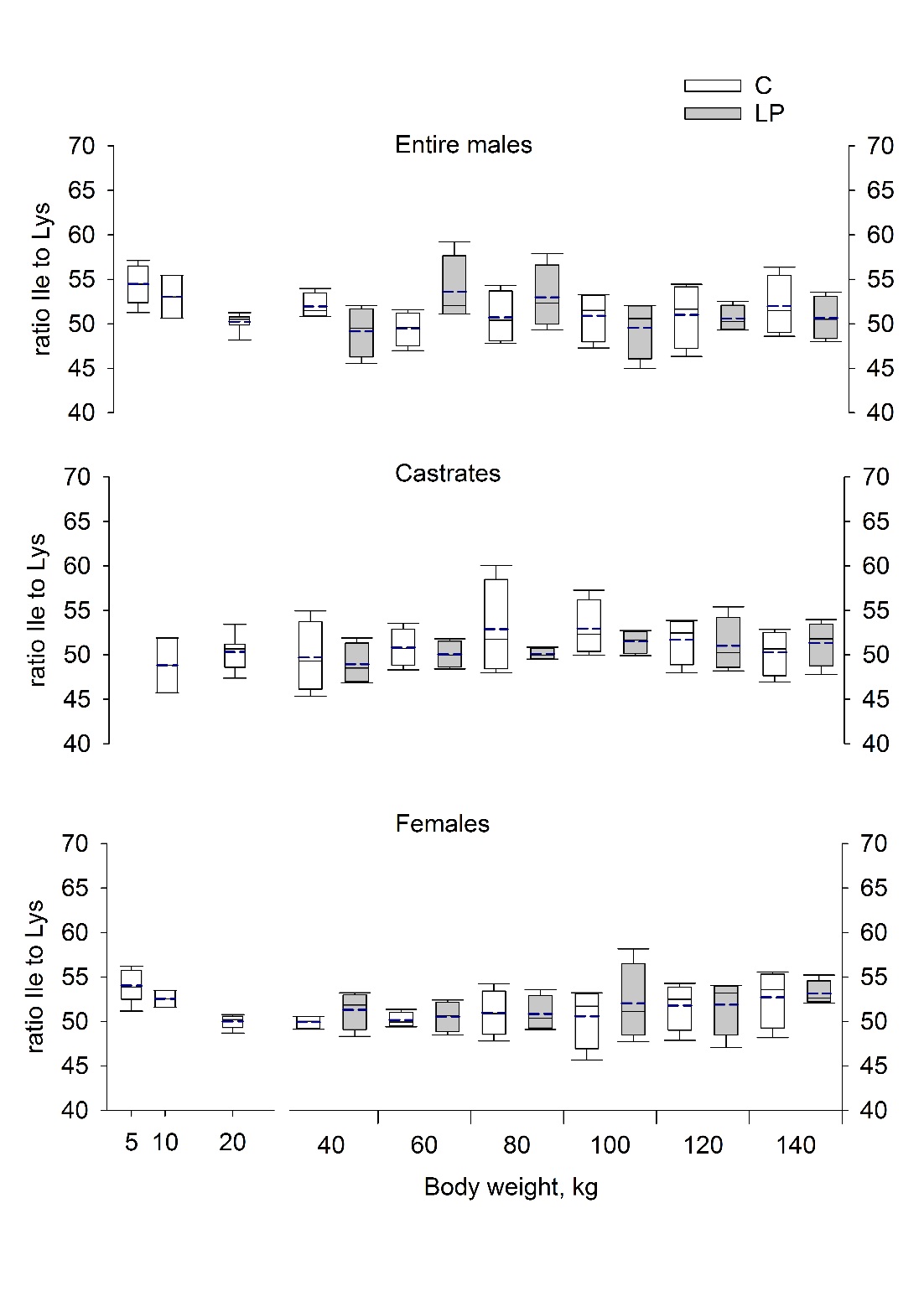


*
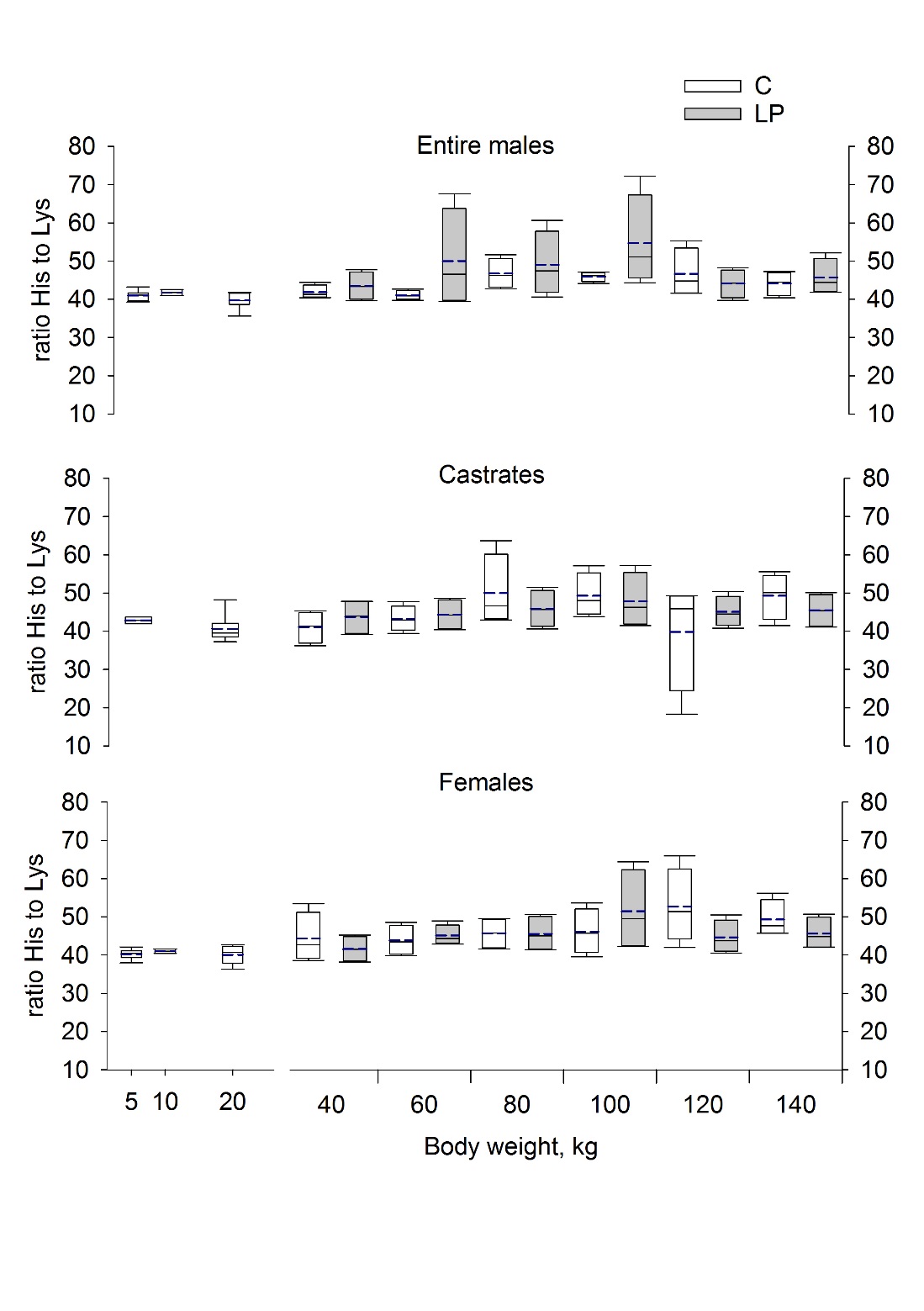
*

Supplementary Figure S1. Changes in the lysine-to-protein and methionine (Met)-, cystine (Cys)-, Met+Cys-, threonine (Thr)-, phenylalanine (Phe)-, tyrosine (Tyr)-, valine (Val)-, leucine (Leu)-, isoleucine (Ile)- and histidine (His)-to-lysine ratios in the empty body of female, castrated and entire male pigs fed the control (C) or low CP (LP) grower (20-60 kg), finisher I (60-100 kg) and finisher II (100-140 kg) diets and slaughtered either the day of birth at 10, 20, 40, 60, 80, 100, 120 or 140 kg BW. Diets C were formulated to meet nutrient requirements according to the standard Swiss feeding recommendations for grower finisher pigs in the respective growth periods; LP diets were formulated to contain, expressed as percentage of the control diets, 80% of dietary CP, lysine, methionine + cystine, threonine and tryptophan. Entire males and FE: n = 8, 2, 8 for the BW categories of new born, 10 and 20 kg; CA pigs: n = 2 and 8 for BW categories of 10 and 20 kg BW. C- and LP-EM, -CA and -FE pigs: n = 4 for the BW categories of 40, 60, 80, 100, 120, 140 kg BW. Box edges represent the upper and lower quantile with median value shown by a solid line and average value by a dashed line, both inside the box. The whiskers represent the 95% confidence interval.

**Supplementary Table S4**. *Relative weight (expressed as percentage of the empty body weight [EBW]) and methionine (Met)-, cystine (Cys)-, threonine (Thr)-, phenylalanine (Phe)-, tyrosine (Tyr)-, valine (Val)-, leucine (Leu)-, isoleucine (Ile)- and histidine (His)-to-Lysine ratios of the carcass fraction of female (FE), castrated (CA) and entire male (EM) pigs fed the control (C) or low CP (LP) grower (20-60 kg), finisher I (60-100 kg) and finisher II (100-140 kg) diets*^1^ *and slaughtered either the day of birth, at 10, 20, 40, 60, 80, 100, 120 or 140 kg BW.*

| BW  category | Diet^1^ | Gender | Relative weight | Protein | Lys: protein | Met | Cys | Thr | Phe | Tyr | Val | Leu | Ile | His |
| --- | --- | --- | --- | --- | --- | --- | --- | --- | --- | --- | --- | --- | --- | --- |
| new born^2^ |  | EM | 74 | 169 | 5.3 | 24.0 | 11.3 | 61.2 | 56.4 | 33.4 | 76.9 | 106.6 | 53.7 | 41.5 |
|  |  | FE | 74 | 164 | 4.7 | 24.4 | 11.5 | 59.5 | 56.5 | 33.0 | 76.8 | 105.0 | 53.1 | 40.4 |
|  |  |  |  |  |  |  |  |  |  |  |  |  |  |  |
| 10^3^ | Starter diet | EM | 77 | 1 150 | 5.3 | 27.2 | 6.9 | 58.7 | 52.9 | 34.3 | 71.1 | 105.0 | 55.7 | 39.9 |
|  |  | CA | 76 | 1 101 | 5.8 | 26.7 | 8.4 | 59.5 | 55.6 | 33.9 | 74.2 | 102.2 | 52.2 | 40.2 |
|  |  | FE | 80 | 972 | 5.8 | 26.9 | 6.2 | 56.3 | 52.9 | 33.3 | 69.7 | 102.5 | 53.7 | 39.1 |
|  |  |  |  |  |  |  |  |  |  |  |  |  |  |  |
| 20 | Starter diet | EM | 74 | 2 245 | 6.6 | 21.5 | 7.3 | 55.4 | 48.4 | 30.9 | 65.2 | 94.5 | 51.9 | 36.5 |
|  |  | CA | 75 | 2 541 | 5.9 | 21.5 | 7.3 | 57.0 | 50.5 | 30.0 | 68.1 | 96.9 | 52.1 | 37.9 |
|  |  | FE | 76 | 3 137 | 6.7 | 19.7 | 6.7 | 54.0 | 48.9 | 31.3 | 65.6 | 94.2 | 51.8 | 37.3 |
|  |  |  |  |  |  |  |  |  |  |  |  |  |  |  |
| 40 | C | EM | 77 | 5 359 | 4.9 | 26.4 | 8.3 | 55.0 | 48.9 | 32.1 | 65.4 | 94.2 | 53.3 | 38.9 |
|  |  | CA | 78 | 4 685 | 7.1 | 20.1 | 7.7 | 56.9 | 49.5 | 32.2 | 66.4 | 93.1 | 50.8 | 38.4 |
|  |  | FE | 79 | 4 907 | 7.4 | 19.2 | 6.4 | 53.9 | 47.7 | 30.5 | 64.1 | 92.3 | 51.6 | 37.7 |
|  | LP | EM | 76 | 4 309 | 6.2 | 24.7 | 6.0 | 54.6 | 52.4 | 33.7 | 65.2 | 94.1 | 50.2 | 39.7 |
|  |  | CA | 77 | 4 533 | 4.9 | 24.2 | 5.5 | 54.1 | 50.4 | 32.0 | 64.4 | 92.1 | 50.0 | 39.9 |
|  |  | FE | 78 | 4 567 | 6.8 | 19.7 | 4.5 | 52.5 | 46.7 | 33.7 | 62.2 | 91.5 | 52.5 | 38.7 |
|  |  |  |  |  |  |  |  |  |  |  |  |  |  |  |
| 60 | C | EM | 79 | 7 812 | 6.7 | 22.4 | 5.0 | 52.8 | 51.1 | 31.1 | 65.5 | 93.8 | 50.6 | 38.5 |
|  |  | CA | 80 | 8 706 | 7.5 | 24.5 | 5.6 | 52.1 | 48.9 | 33.2 | 63.8 | 91.8 | 51.6 | 40.9 |
|  |  | FE | 81 | 8 384 | 7.6 | 23.6 | 4.8 | 53.6 | 47.2 | 31.1 | 63.4 | 92.8 | 52.1 | 41.8 |
|  | LP | EM | 79 | 7 004 | 6.5 | 19.5 | 5.1 | 62.4 | 51.5 | 36.9 | 69.4 | 100.8 | 56.1 | 49.6 |
|  |  | CA | 80 | 6 549 | 5.9 | 23.4 | 4.9 | 53.7 | 48.2 | 33.9 | 64.1 | 93.1 | 51.4 | 42.2 |
|  |  | FE | 81 | 7 423 | 3.4 | 24.3 | 5.5 | 55.0 | 51.4 | 32.6 | 66.1 | 93.6 | 51.9 | 42.2 |
|  |  |  |  |  |  |  |  |  |  |  |  |  |  |  |
| 80 | C | EM | 80 | 11 863 | 6.6 | 19.0 | 4.7 | 54.4 | 47.0 | 32.4 | 63.9 | 92.2 | 51.8 | 45.1 |
|  |  | CA | 81 | 10 692 | 7.6 | 16.9 | 4.8 | 56.9 | 47.7 | 35.5 | 65.3 | 95.7 | 55.0 | 47.8 |
|  |  | FE | 81 | 11 412 | 6.0 | 25.9 | 5.8 | 52.0 | 49.3 | 33.1 | 65.2 | 93.4 | 52.8 | 42.9 |
|  | LP | EM | 79 | 11 043 | 6.4 | 19.2 | 4.9 | 58.8 | 47.6 | 32.6 | 65.3 | 96.7 | 54.2 | 48.3 |
|  |  | CA | 81 | 9 688 | 5.4 | 20.9 | 5.0 | 52.8 | 46.0 | 31.5 | 62.4 | 90.9 | 51.1 | 43.3 |
|  |  | FE | 81 | 9 624 | 7.6 | 23.2 | 5.5 | 55.0 | 49.6 | 34.3 | 65.9 | 94.9 | 52.6 | 43.7 |
|  |  |  |  |  |  |  |  |  |  |  |  |  |  |  |
| 100 | C | EM | 81 | 13 302 | 7.5 | 21.3 | 4.7 | 55.4 | 48.7 | 33.4 | 65.3 | 93.0 | 52.1 | 43.2 |
|  |  | CA | 83 | 12 901 | 5.7 | 21.7 | 5.9 | 57.2 | 50.0 | 32.5 | 66.8 | 98.0 | 55.1 | 48.4 |
|  |  | FE | 83 | 14 282 | 7.1 | 21.1 | 4.6 | 52.6 | 47.5 | 33.3 | 64.3 | 90.4 | 51.6 | 44.8 |
|  | LP | EM | 80 | 12 739 | 5.4 | 22.9 | 4.7 | 52.7 | 47.6 | 30.6 | 63.8 | 93.2 | 51.5 | 44.9 |
|  |  | CA | 82 | 12 964 | 7.4 | 25.8 | 6.0 | 54.9 | 48.4 | 34.3 | 63.9 | 95.3 | 52.9 | 46.6 |
|  |  | FE | 83 | 13 645 | 6.7 | 17.1 | 4.4 | 57.1 | 47.9 | 32.1 | 62.7 | 96.6 | 53.4 | 50.4 |
|  |  |  |  |  |  |  |  |  |  |  |  |  |  |  |
| 120 | C | EM | 81 | 17 763 | 6.9 | 16.3 | 5.8 | 55.8 | 47.1 | 33.0 | 64.0 | 93.4 | 52.5 | 44.5 |
|  |  | CA | 84 | 15 751 | 7.5 | 22.9 | 5.8 | 57.6 | 50.2 | 33.9 | 66.2 | 103.5 | 52.4 | 35.2 |
|  |  | FE | 83 | 15 024 | 6.4 | 23.8 | 4.7 | 54.4 | 48.4 | 33.3 | 63.7 | 94.0 | 54.0 | 45.7 |
|  | LP | EM | 81 | 15 756 | 6.8 | 20.0 | 5.1 | 51.3 | 45.8 | 31.2 | 61.9 | 91.7 | 51.3 | 42.3 |
|  |  | CA | 84 | 16 356 | 6.9 | 24.2 | 5.6 | 50.9 | 46.6 | 33.8 | 62.8 | 92.1 | 52.5 | 43.4 |
|  |  | FE | 83 | 15 174 | 6.7 | 23.9 | 5.9 | 48.8 | 45.4 | 33.5 | 60.7 | 93.2 | 52.9 | 42.5 |
|  |  |  |  |  |  |  |  |  |  |  |  |  |  |  |
| 140 | C | EM | 82 | 19 842 | 7.3 | 21.5 | 5.5 | 54.4 | 48.7 | 33.9 | 65.9 | 93.8 | 53.1 | 42.1 |
|  |  | CA | 84 | 17 758 | 6.5 | 20.7 | 5.4 | 51.6 | 48.8 | 31.9 | 63.3 | 94.3 | 51.7 | 48.2 |
|  |  | FE | 83 | 18 654 | 3.6 | 23.5 | 5.8 | 52.8 | 46.9 | 32.7 | 62.3 | 97.3 | 54.2 | 48.0 |
|  | LP | EM | 81 | 17 098 | 6.1 | 19.9 | 5.4 | 51.9 | 46.4 | 32.6 | 61.8 | 91.8 | 51.9 | 43.9 |
|  |  | CA | 84 | 17 913 | 6.2 | 22.6 | 7.1 | 49.6 | 46.7 | 32.4 | 62.9 | 92.9 | 52.6 | 43.4 |
|  |  | FE | 84 | 18 456 | 9.1 | 23.7 | 6.0 | 52.2 | 47.9 | 33.1 | 65.6 | 95.1 | 53.8 | 44.0 |

^1^ Control diets (C) were formulated to meet nutrient requirements according to the standard Swiss feeding recommendations for grower finisher pigs in the respective growth periods; low protein diets (LP) were formulated to contain, expressed as percentage of the control diets, 80% of dietary CP, lysine, methionine + cystine, threonine and tryptophan.

^2^ New born piglets were not separated into the five different fractions. Thus, results presented in this table refer to skin and hooves, carcass and blood.

^3^ Skin and claws were not removed from the carcass of piglets slaughtered at 10 kg BW. Thus, the results presented in this table for this category refer to the carcass and skin and claws fraction.

**Supplementary Table S5**. *Relative weight (expressed as percentage of the empty body weight [EBW]) and methionine (Met)-, cystine (Cys)-, threonine (Thr)-, phenylalanine (Phe)-, tyrosine (Tyr)-, valine (Val)-, leucine (Leu)-, isoleucine (Ile)- and histidine (His)-to-Lysine ratios of the organs and intestines fraction of female (FE), castrated (CA) and entire male (EM) pigs fed the control (C) or low CP (LP) grower (20-60 kg), finisher I (60-100 kg) and finisher II (100-140 kg) diets*^1^ *and slaughtered either the day of birth, at 10, 20, 40, 60, 80, 100, 120 or 140 kg BW.*

| BW  category | Diet^1^ | Gender | Relative weight | Protein | Lys: protein | Met | Cys | Thr | Phe | Tyr | Val | Leu | Ile | His |
| --- | --- | --- | --- | --- | --- | --- | --- | --- | --- | --- | --- | --- | --- | --- |
| new born |  | EM | 18 | 36 | 5.9 | 24.8 | 6.8 | 62.2 | 59.5 | 32.7 | 80.3 | 113.4 | 57.7 | 38.5 |
|  |  | FE | 18 | 37 | 5.0 | 24.5 | 6.3 | 66.0 | 59.5 | 30.9 | 83.1 | 115.3 | 58.1 | 39.7 |
|  |  |  |  |  |  |  |  |  |  |  |  |  |  |  |
| 10 | Starter diet | EM | 17 | 155 | 6.4 | 24.9 | 6.2 | 55.8 | 57.1 | 39.6 | 71.7 | 107.5 | 53.1 | 36.3 |
|  |  | CA | 17 | 217 | 5.7 | 25.0 | 6.4 | 56.5 | 57.2 | 40.2 | 72.2 | 109.3 | 53.7 | 36.3 |
|  |  | FE | 16 | 182 | 6.2 | 26.4 | 7.1 | 60.0 | 60.9 | 40.0 | 79.0 | 114.3 | 57.1 | 38.6 |
|  |  |  |  |  |  |  |  |  |  |  |  |  |  |  |
| 20 | Starter diet | EM | 18 | 425 | 6.7 | 25.9 | 10.7 | 60.8 | 60.5 | 40.4 | 81.2 | 113.6 | 57.5 | 36.3 |
|  |  | CA | 18 | 574 | 6.9 | 25.9 | 11.4 | 60.7 | 59.4 | 39.9 | 79.2 | 112.5 | 57.0 | 36.3 |
|  |  | FE | 17 | 762 | 7.0 | 33.0 | 30.5 | 69.4 | 59.4 | 39.2 | 70.4 | 117.5 | 60.5 | 43.7 |
|  |  |  |  |  |  |  |  |  |  |  |  |  |  |  |
| 40 | C | EM | 17 | 1 092 | 4.3 | 26.2 | 9.4 | 62.3 | 63.3 | 42.7 | 84.5 | 118.3 | 59.0 | 38.1 |
|  |  | CA | 15 | 727 | 5.3 | 24.8 | 10.0 | 59.8 | 60.9 | 40.9 | 79.4 | 112.1 | 56.1 | 36.2 |
|  |  | FE | 15 | 774 | 4.9 | 26.6 | 8.9 | 61.3 | 61.5 | 40.4 | 78.9 | 112.1 | 53.3 | 85.7 |
|  | LP | EM | 16 | 795 | 5.0 | 24.4 | 12.4 | 62.1 | 60.8 | 40.2 | 81.8 | 113.2 | 56.9 | 36.7 |
|  |  | CA | 16 | 762 | 5.0 | 23.3 | 11.9 | 60.4 | 59.0 | 37.2 | 78.6 | 111.4 | 56.0 | 36.2 |
|  |  | FE | 15 | 741 | 4.9 | 26.6 | 8.2 | 63.1 | 66.9 | 43.5 | 90.2 | 118.3 | 57.3 | 37.7 |
|  |  |  |  |  |  |  |  |  |  |  |  |  |  |  |
| 60 | C | EM | 14 | 1 208 | 7.9 | 23.5 | 11.9 | 61.0 | 59.4 | 35.9 | 78.9 | 111.8 | 55.3 | 38.0 |
|  |  | CA | 14 | 1 191 | 7.3 | 25.9 | 12.2 | 63.9 | 60.2 | 40.1 | 79.4 | 112.8 | 57.5 | 38.7 |
|  |  | FE | 13 | 1 102 | 7.6 | 23.0 | 8.8 | 59.8 | 59.8 | 40.3 | 74.6 | 110.2 | 54.4 | 36.5 |
|  | LP | EM | 15 | 1 251 | 4.9 | 24.1 | 7.7 | 63.3 | 63.0 | 38.1 | 83.4 | 111.3 | 54.7 | 39.4 |
|  |  | CA | 14 | 1 063 | 8.4 | 23.9 | 9.9 | 62.4 | 59.6 | 37.3 | 76.4 | 110.5 | 56.0 | 37.5 |
|  |  | FE | 13 | 910 | 7.7 | 24.0 | 11.8 | 65.0 | 64.1 | 40.6 | 88.9 | 116.4 | 57.8 | 39.1 |
|  |  |  |  |  |  |  |  |  |  |  |  |  |  |  |
| 80 | C | EM | 14 | 1 573 | 6.8 | 24.1 | 9.3 | 66.6 | 61.1 | 41.3 | 79.8 | 113.7 | 57.5 | 39.1 |
|  |  | CA | 13 | 1 359 | 5.1 | 22.9 | 10.1 | 61.8 | 60.3 | 37.7 | 79.8 | 113.6 | 55.8 | 39.4 |
|  |  | FE | 13 | 1 536 | 5.9 | 23.1 | 8.4 | 59.2 | 58.8 | 40.0 | 77.4 | 110.6 | 55.1 | 36.6 |
|  | LP | EM | 14 | 1 423 | 5.7 | 20.2 | 7.2 | 64.1 | 61.2 | 38.5 | 79.6 | 115.3 | 57.2 | 39.4 |
|  |  | CA | 13 | 1 142 | 5.7 | 23.1 | 8.5 | 62.6 | 62.3 | 39.4 | 83.1 | 118.0 | 57.8 | 39.2 |
|  |  | FE | 13 | 1 538 | 5.9 | 26.0 | 9.8 | 62.3 | 60.4 | 39.1 | 78.2 | 111.3 | 55.9 | 38.0 |
|  |  |  |  |  |  |  |  |  |  |  |  |  |  |  |
| 100 | C | EM | 13 | 1 940 | 6.0 | 22.2 | 9.9 | 59.0 | 57.3 | 34.3 | 75.4 | 108.9 | 54.4 | 37.2 |
|  |  | CA | 12 | 1 538 | 4.8 | 21.3 | 8.8 | 61.9 | 59.2 | 36.7 | 78.7 | 112.0 | 55.2 | 36.9 |
|  |  | FE | 12 | 1 508 | 6.5 | 24.2 | 7.6 | 60.9 | 59.5 | 38.4 | 79.5 | 114.1 | 57.9 | 37.2 |
|  | LP | EM | 14 | 1 718 | 3.9 | 18.1 | 7.4 | 66.1 | 58.5 | 40.2 | 78.4 | 115.7 | 54.3 | 119.2 |
|  |  | CA | 12 | 1 561 | 6.1 | 20.5 | 9.0 | 59.2 | 58.4 | 41.2 | 75.8 | 113.2 | 56.4 | 35.1 |
|  |  | FE | 12 | 1 389 | 4.8 | 20.6 | 8.1 | 66.0 | 66.4 | 41.1 | 94.4 | 119.4 | 59.0 | 39.7 |
|  |  |  |  |  |  |  |  |  |  |  |  |  |  |  |
| 120 | C | EM | 13 | 1 276 | 6.2 | 23.3 | 8.5 | 59.3 | 61.5 | 40.8 | 79.8 | 114.3 | 56.6 | 38.9 |
|  |  | CA | 11 | 1 615 | 6.3 | 23.2 | 10.1 | 62.5 | 62.9 | 40.5 | 81.6 | 119.7 | 58.3 | 39.7 |
|  |  | FE | 12 | 1 812 | 5.4 | 21.9 | 8.0 | 62.1 | 57.9 | 39.8 | 72.6 | 110.2 | 52.5 | 92.8 |
|  | LP | EM | 13 | 1 911 | 7.3 | 24.1 | 10.4 | 61.8 | 61.5 | 41.7 | 81.3 | 114.9 | 58.3 | 37.8 |
|  |  | CA | 11 | 1 478 | 9.8 | 19.7 | 8.7 | 58.7 | 60.7 | 40.5 | 79.2 | 114.6 | 56.1 | 37.1 |
|  |  | FE | 12 | 2 740 | 6.0 | 21.4 | 7.4 | 61.6 | 62.6 | 39.8 | 77.6 | 120.4 | 56.4 | 39.3 |
|  |  |  |  |  |  |  |  |  |  |  |  |  |  |  |
| 140 | C | EM | 12 | 2 172 | 7.7 | 23.6 | 9.0 | 62.7 | 61.9 | 40.1 | 80.7 | 116.4 | 58.2 | 39.7 |
|  |  | CA | 11 | 1 701 | 4.5 | 21.1 | 9.9 | 59.5 | 66.0 | 37.0 | 82.8 | 119.7 | 55.7 | 39.0 |
|  |  | FE | 11 | 2 006 | 6.3 | 19.3 | 7.7 | 64.2 | 62.5 | 36.6 | 84.5 | 116.8 | 55.9 | 39.7 |
|  | LP | EM | 13 | 2 179 | 6.5 | 21.6 | 8.3 | 62.4 | 60.8 | 38.7 | 75.8 | 118.7 | 55.9 | 38.0 |
|  |  | CA | 11 | 1 824 | 6.5 | 19.9 | 7.7 | 63.8 | 65.5 | 38.2 | 76.8 | 131.8 | 58.0 | 39.7 |
|  |  | FE | 11 | 1 932 | 5.4 | 25.0 | 11.0 | 60.6 | 64.0 | 44.7 | 86.0 | 121.7 | 59.2 | 39.9 |

^1^ Control diets (C) were formulated to meet nutrient requirements according to the standard Swiss feeding recommendations for grower finisher pigs in the respective growth periods; low protein diets (LP) were formulated to contain, expressed as percentage of the control diets, 80% of dietary CP, lysine, methionine + cystine, threonine and tryptophan.

**Supplementary Table S6**. *Relative weight (expressed as percentage of the empty body weight [EBW]) and methionine (Met)-, cystine (Cys)-, threonine (Thr)-, phenylalanine (Phe)-, tyrosine (Tyr)-, valine (Val)-, leucine (Leu)-, isoleucine (Ile)- and histidine (His)-to-Lysine ratios of the blood fraction of female (FE), castrated (CA) and entire male (EM) pigs fed the control (C) or low CP (LP) grower (20-60 kg), finisher I (60-100 kg) and finisher II (100-140 kg) diets*^1^ *and slaughtered either the day of birth, at 10, 20, 40, 60, 80, 100, 120 or 140 kg BW.*

| BW  category | Diet^1^ | Gender | Relative weight | Protein | Lys: protein | Met | Cys | Thr | Phe | Tyr | Val | Leu | Ile | His |
| --- | --- | --- | --- | --- | --- | --- | --- | --- | --- | --- | --- | --- | --- | --- |
| new born^2^ |  | EM |  |  |  |  |  |  |  |  |  |  |  |  |
|  |  | FE |  |  |  |  |  |  |  |  |  |  |  |  |
|  |  |  |  |  |  |  |  |  |  |  |  |  |  |  |
| 10 | Starter diet | EM | 6.4 | 78 | 5.6 | 8.4 | 10.7 | 42.8 | 76.6 | 29.2 | 101.0 | 150.8 | 14.2 | 81.2 |
|  |  | CA | 7.0 | 103 | 7.7 | 7.9 | 10.7 | 39.2 | 72.4 | 26.4 | 93.1 | 140.5 | 11.8 | 77.5 |
|  |  | FE | 4.1 | 81 | 6.0 | 8.5 | 9.0 | 44.9 | 79.9 | 29.4 | 106.0 | 160.2 | 12.4 | 88.0 |
|  |  |  |  |  |  |  |  |  |  |  |  |  |  |  |
| 20 | Starter diet | EM | 6.7 | 164 | 6.8 | 8.7 | 10.8 | 42.7 | 74.6 | 25.2 | 101.9 | 144.4 | 13.7 | 78.7 |
|  |  | CA | 6.5 | 184 | 8.8 | 8.5 | 12.9 | 42.7 | 74.4 | 25.9 | 99.8 | 143.2 | 14.1 | 78.6 |
|  |  | FE | 6.5 | 224 | 7.7 | 8.5 | 11.0 | 42.6 | 74.4 | 26.0 | 100.7 | 144.7 | 13.0 | 78.7 |
|  |  |  |  |  |  |  |  |  |  |  |  |  |  |  |
| 40 | C | EM | 5.4 | 403 | 5.9 | 8.8 | 10.3 | 44.4 | 79.0 | 28.7 | 107.0 | 154.7 | 14.1 | 86.5 |
|  |  | CA | 5.5 | 330 | 6.0 | 8.7 | 11.2 | 42.6 | 75.3 | 25.3 | 99.9 | 144.8 | 15.3 | 77.9 |
|  |  | FE | 5.4 | 390 | 6.1 | 8.0 | 9.7 | 41.6 | 75.0 | 25.1 | 99.6 | 144.6 | 13.0 | 89.1 |
|  | LP | EM | 5.6 | 299 | 6.5 | 8.3 | 12.0 | 45.0 | 73.3 | 24.6 | 99.6 | 141.5 | 15.4 | 78.9 |
|  |  | CA | 4.6 | 333 | 6.2 | 8.3 | 11.2 | 43.8 | 73.9 | 25.5 | 100.3 | 143.1 | 15.1 | 87.9 |
|  |  | FE | 5.1 | 348 | 5.7 | 8.0 | 8.3 | 43.6 | 74.9 | 24.7 | 102.3 | 145.8 | 14.2 | 90.9 |
|  |  |  |  |  |  |  |  |  |  |  |  |  |  |  |
| 60 | C | EM | 5.0 | 574 | 9.4 | 7.6 | 11.6 | 43.3 | 72.1 | 23.3 | 97.5 | 139.4 | 14.5 | 77.4 |
|  |  | CA | 4.6 | 695 | 7.7 | 8.8 | 13.6 | 46.2 | 78.6 | 27.4 | 102.1 | 144.7 | 15.2 | 84.2 |
|  |  | FE | 5.1 | 633 | 9.1 | 8.4 | 9.5 | 42.7 | 75.0 | 26.7 | 97.6 | 141.8 | 14.1 | 78.2 |
|  | LP | EM | 5.1 | 584 | 5.6 | 8.7 | 8.7 | 43.6 | 74.5 | 26.3 | 99.8 | 142.3 | 14.7 | 80.2 |
|  |  | CA | 5.0 | 476 | 9.2 | 8.6 | 11.5 | 44.8 | 75.1 | 27.0 | 98.6 | 141.4 | 16.0 | 77.8 |
|  |  | FE | 4.6 | 396 | 9.2 | 8.5 | 11.8 | 44.6 | 74.8 | 25.9 | 100.1 | 143.5 | 14.6 | 80.0 |
|  |  |  |  |  |  |  |  |  |  |  |  |  |  |  |
| 80 | C | EM | 5.0 | 795 | 7.3 | 8.5 | 10.6 | 44.7 | 77.8 | 26.6 | 103.8 | 149.3 | 15.0 | 84.6 |
|  |  | CA | 4.7 | 778 | 6.7 | 7.8 | 9.9 | 43.3 | 75.7 | 24.5 | 100.0 | 144.9 | 14.6 | 83.4 |
|  |  | FE | 5.0 | 695 | 9.1 | 7.0 | 9.9 | 44.3 | 77.7 | 27.8 | 102.9 | 149.5 | 15.9 | 83.4 |
|  | LP | EM | 4.8 | 659 | 4.7 | 7.7 | 8.2 | 44.7 | 76.4 | 26.4 | 102.1 | 146.5 | 15.3 | 83.1 |
|  |  | CA | 4.4 | 623 | 7.4 | 7.4 | 7.6 | 43.3 | 73.6 | 22.6 | 99.5 | 141.9 | 14.6 | 91.3 |
|  |  | FE | 4.7 | 707 | 8.0 | 8.2 | 9.2 | 41.9 | 74.6 | 25.5 | 98.5 | 141.9 | 13.6 | 81.7 |
|  |  |  |  |  |  |  |  |  |  |  |  |  |  |  |
| 100 | C | EM | 4.5 | 836 | 6.9 | 6.2 | 8.1 | 40.8 | 72.7 | 21.7 | 96.1 | 139.2 | 15.2 | 79.7 |
|  |  | CA | 4.4 | 997 | 8.1 | 8.0 | 9.4 | 41.8 | 73.9 | 25.0 | 96.8 | 139.0 | 15.5 | 77.6 |
|  |  | FE | 4.4 | 895 | 7.1 | 7.7 | 8.0 | 40.2 | 72.5 | 23.5 | 96.9 | 137.9 | 14.7 | 79.4 |
|  | LP | EM | 5.1 | 947 | 6.9 | 10.0 | 8.4 | 45.3 | 80.0 | 26.8 | 110.4 | 154.8 | 15.6 | 97.4 |
|  |  | CA | 4.4 | 959 | 6.1 | 7.7 | 9.3 | 45.3 | 78.1 | 27.3 | 105.0 | 149.3 | 16.0 | 87.2 |
|  |  | FE | 4.3 | 737 | 8.0 | 7.7 | 8.1 | 45.6 | 77.6 | 27.1 | 104.2 | 147.8 | 15.7 | 87.3 |
|  |  |  |  |  |  |  |  |  |  |  |  |  |  |  |
| 120 | C | EM | 4.6 | 1 086 | 6.1 | 8.6 | 8.6 | 45.2 | 79.1 | 27.2 | 106.5 | 151.0 | 14.1 | 87.1 |
|  |  | CA | 3.9 | 1 035 | 8.0 | 7.7 | 7.9 | 43.3 | 75.5 | 25.5 | 98.2 | 141.9 | 15.0 | 83.3 |
|  |  | FE | 4.4 | 1 152 | 5.1 | 6.6 | 7.3 | 42.0 | 75.8 | 25.4 | 94.7 | 144.6 | 13.3 | 98.4 |
|  | LP | EM | 4.3 | 863 | 8.7 | 7.9 | 9.3 | 42.0 | 75.2 | 26.4 | 98.8 | 142.5 | 14.0 | 81.5 |
|  |  | CA | 4.0 | 1 051 | 6.9 | 7.4 | 9.3 | 41.1 | 78.0 | 26.5 | 102.3 | 150.6 | 13.1 | 82.8 |
|  |  | FE | 4.2 | 970 | 8.2 | 6.8 | 7.8 | 46.3 | 77.1 | 26.0 | 101.8 | 147.8 | 17.1 | 87.9 |
|  |  |  |  |  |  |  |  |  |  |  |  |  |  |  |
| 140 | C | EM | 4.4 | 1 205 | 9.2 | 8.2 | 8.6 | 43.0 | 74.7 | 25.4 | 99.8 | 141.0 | 16.7 | 81.1 |
|  |  | CA | 3.8 | 1 205 | 8.2 | 7.6 | 8.2 | 38.7 | 73.8 | 24.3 | 96.8 | 140.7 | 12.7 | 80.4 |
|  |  | FE | 4.1 | 1 281 | 5.8 | 7.4 | 8.3 | 43.5 | 76.3 | 24.2 | 101.2 | 145.1 | 16.5 | 83.6 |
|  | LP | EM | 4.3 | 1 383 | 8.1 | 7.1 | 8.0 | 43.4 | 73.2 | 25.1 | 96.1 | 138.3 | 17.5 | 80.8 |
|  |  | CA | 3.8 | 1 204 | 5.4 | 7.5 | 8.3 | 47.0 | 77.8 | 26.5 | 103.9 | 147.2 | 17.2 | 87.0 |
|  |  | FE | 3.7 | 1 254 | 8.7 | 6.6 | 8.5 | 41.5 | 76.6 | 26.5 | 99.4 | 146.5 | 13.2 | 82.7 |

^1^Control diets (C) were formulated to meet nutrient requirement according to the standard Swiss feeding recommendations for grower finisher pigs in the respective growth periods; low protein diets (LP) were formulated to contain, expressed as percentage of the control diets, 80% of dietary CP, lysine, methionine + cystine, threonine and tryptophan.

^2^New born piglets were not separated into the five different fractions. Results are presented in Supplementary Tables S4 and S5.

**Supplementary Table S7**. *Relative weight (expressed as percentage of the empty body weight [EBW]) and methionine (Met)-, cystine (Cys)-, threonine (Thr)-, phenylalanine (Phe)-, tyrosine (Tyr)-, valine (Val)-, leucine (Leu)-, isoleucine (Ile)- and histidine (His)-to-Lysine ratios of the skin and claws fraction of female (FE), castrated (CA) and entire male (EM) pigs fed the control (C) or low CP (LP) grower (20-60 kg), finisher I (60-100 kg) and finisher II (100-140 kg) diets*^1^ *and slaughtered either the day of birth, at 10, 20, 40, 60, 80, 100, 120 or 140 kg BW.*

| BW  category | Diet^1^ | Gender | Relative weight | Protein | Lys: protein | Met | Cys | Thr | Phe | Tyr | Val | Leu | Ile | His |
| --- | --- | --- | --- | --- | --- | --- | --- | --- | --- | --- | --- | --- | --- | --- |
| new born^2^ |  | EM |  |  |  |  |  |  |  |  |  |  |  |  |
|  |  | FE |  |  |  |  |  |  |  |  |  |  |  |  |
|  |  |  |  |  |  |  |  |  |  |  |  |  |  |  |
| 10^3^ | Starter diet | EM |  |  |  |  |  |  |  |  |  |  |  |  |
|  |  | CA |  |  |  |  |  |  |  |  |  |  |  |  |
|  |  | FE |  |  |  |  |  |  |  |  |  |  |  |  |
|  |  |  |  |  |  |  |  |  |  |  |  |  |  |  |
| 20 | Starter diet | EM | 0.1 | 53 | 4.8 | 22.1 | 127.3 | 93.1 | 71.6 | 69.6 | 108.2 | 163.0 | 78.6 | 39.1 |
|  |  | CA | 0.1 | 62 | 5.3 | 22.2 | 141.5 | 94.1 | 70.2 | 71.0 | 106.4 | 160.4 | 77.7 | 40.7 |
|  |  | FE | 0.1 | 61 | 4.2 | 21.8 | 152.5 | 95.2 | 70.6 | 68.7 | 109.2 | 161.5 | 79.5 | 39.3 |
|  |  |  |  |  |  |  |  |  |  |  |  |  |  |  |
| 40 | C | EM | 0.1 | 83 | 3.6 | 19.7 | 178.1 | 101.4 | 72.6 | 78.2 | 116.5 | 172.4 | 82.4 | 40.2 |
|  |  | CA | 0.1 | 155 | 3.8 | 19.6 | 180.2 | 109.1 | 71.5 | 77.5 | 117.2 | 176.1 | 80.9 | 119.7 |
|  |  | FE | 0.1 | 96 | 3.7 | 17.2 | 149.6 | 99.0 | 72.3 | 75.1 | 112.7 | 172.1 | 80.9 | 41.7 |
|  | LP | EM | 0.1 | 175 | 3.3 | 19.5 | 138.4 | 102.2 | 71.6 | 73.1 | 113.1 | 172.2 | 78.8 | 122.9 |
|  |  | CA | 0.1 | 75 | 3.4 | 21.1 | 135.3 | 100.8 | 73.4 | 77.7 | 112.6 | 173.7 | 76.6 | 118.4 |
|  |  | FE | 0.1 | 183 | 3.2 | 15.3 | 143.1 | 102.4 | 72.2 | 69.6 | 115.3 | 168.3 | 79.3 | 44.5 |
|  |  |  |  |  |  |  |  |  |  |  |  |  |  |  |
| 60 | C | EM | 0.1 | 223 | 5.0 | 18.5 | 229.2 | 111.0 | 71.2 | 69.0 | 123.3 | 175.7 | 83.9 | 38.6 |
|  |  | CA | 0.1 | 248 | 4.2 | 19.7 | 153.9 | 107.4 | 71.8 | 81.5 | 120.4 | 178.6 | 83.1 | 38.9 |
|  |  | FE | 0.1 | 154 | 3.6 | 20.7 | 155.6 | 107.4 | 75.5 | 91.6 | 124.4 | 179.7 | 84.6 | 37.9 |
|  | LP | EM | 0.1 | 206 | 3.4 | 20.0 | 143.5 | 109.9 | 73.8 | 85.6 | 124.3 | 180.1 | 85.9 | 42.3 |
|  |  | CA | 0.1 | 299 | 5.0 | 19.6 | 167.4 | 109.4 | 72.1 | 71.5 | 117.4 | 172.4 | 81.9 | 40.6 |
|  |  | FE | 0.1 | 275 | 4.3 | 20.1 | 147.4 | 100.9 | 73.5 | 83.3 | 113.7 | 170.1 | 80.8 | 42.0 |
|  |  |  |  |  |  |  |  |  |  |  |  |  |  |  |
| 80 | C | EM | 0.1 | 320 | 3.3 | 20.5 | 183.3 | 120.7 | 75.4 | 96.4 | 135.4 | 190.5 | 88.9 | 38.5 |
|  |  | CA | 0.1 | 330 | 3.6 | 19.3 | 129.5 | 102.0 | 71.1 | 74.8 | 115.5 | 175.3 | 79.4 | 112.5 |
|  |  | FE | 0.1 | 596 | 4.6 | 14.9 | 177.9 | 113.4 | 70.5 | 74.0 | 130.0 | 183.4 | 86.3 | 37.6 |
|  | LP | EM | 0.1 | 305 | 4.4 | 18.2 | 140.2 | 105.0 | 74.2 | 84.1 | 119.3 | 175.6 | 85.6 | 43.8 |
|  |  | CA | 0.1 | 416 | 3.6 | 13.8 | 139.3 | 106.5 | 72.0 | 79.9 | 123.1 | 181.9 | 84.2 | 37.9 |
|  |  | FE | 0.1 | 322 | 4.3 | 20.8 | 145.7 | 108.3 | 72.8 | 86.6 | 122.0 | 179.5 | 85.7 | 42.1 |
|  |  |  |  |  |  |  |  |  |  |  |  |  |  |  |
| 100 | C | EM | 0.1 | 698 | 3.7 | 15.1 | 158.2 | 108.2 | 70.3 | 84.1 | 119.6 | 179.2 | 82.2 | 107.6 |
|  |  | CA | 0.1 | 474 | 4.5 | 18.9 | 191.2 | 109.9 | 73.0 | 77.8 | 126.0 | 177.1 | 87.3 | 38.6 |
|  |  | FE | 0.1 | 533 | 3.5 | 19.5 | 170.3 | 109.0 | 73.5 | 85.1 | 129.4 | 184.6 | 89.8 | 36.4 |
|  | LP | EM | 0.1 | 285 | 4.7 | 20.0 | 121.2 | 101.7 | 69.5 | 84.8 | 111.3 | 169.8 | 79.3 | 39.0 |
|  |  | CA | 0.1 | 423 | 4.2 | 17.3 | 156.0 | 104.0 | 72.9 | 79.2 | 121.0 | 174.6 | 84.4 | 38.6 |
|  |  | FE | 0.1 | 741 | 4.2 | 17.9 | 159.0 | 114.3 | 72.5 | 85.7 | 128.0 | 181.0 | 87.5 | 41.8 |
|  |  |  |  |  |  |  |  |  |  |  |  |  |  |  |
| 120 | C | EM | 0.1 | 630 | 4.5 | 21.1 | 116.5 | 109.9 | 70.2 | 91.3 | 117.1 | 171.2 | 80.9 | 43.1 |
|  |  | CA | 0.1 | 624 | 4.9 | 18.7 | 150.0 | 111.3 | 73.4 | 93.8 | 124.3 | 180.0 | 87.0 | 39.8 |
|  |  | FE | 0.1 | 722 | 3.4 | 11.9 | 152.1 | 106.8 | 71.2 | 82.8 | 119.1 | 176.1 | 85.7 | 41.7 |
|  | LP | EM | 0.1 | 756 | 4.4 | 19.6 | 137.8 | 110.2 | 77.2 | 93.2 | 124.3 | 184.5 | 88.7 | 42.0 |
|  |  | CA | 0.1 | 529 | 3.7 | 15.6 | 173.1 | 110.1 | 74.5 | 79.7 | 127.2 | 184.8 | 87.6 | 40.1 |
|  |  | FE | 0.1 | 711 | 4.7 | 18.6 | 123.7 | 97.8 | 73.9 | 80.0 | 113.0 | 172.1 | 83.3 | 39.4 |
|  |  |  |  |  |  |  |  |  |  |  |  |  |  |  |
| 140 | C | EM | 0.1 | 521 | 4.3 | 23.8 | 121.9 | 111.8 | 70.7 | 90.5 | 121.0 | 179.3 | 82.5 | 38.2 |
|  |  | CA | 0.1 | 650 | 4.9 | 21.5 | 126.8 | 96.3 | 73.1 | 84.7 | 115.1 | 177.1 | 83.9 | 35.3 |
|  |  | FE | 0.1 | 803 | 3.9 | 18.3 | 139.7 | 99.7 | 73.0 | 89.6 | 116.2 | 172.7 | 84.6 | 38.6 |
|  | LP | EM | 0.1 | 561 | 4.8 | 18.6 | 168.9 | 109.7 | 74.6 | 89.2 | 126.9 | 182.8 | 87.1 | 41.2 |
|  |  | CA | 0.1 | 605 | 3.7 | 19.7 | 164.5 | 109.2 | 74.9 | 99.8 | 123.4 | 181.9 | 87.4 | 41.8 |
|  |  | FE | 0.1 | 960 | 4.6 | 12.4 | 172.7 | 118.9 | 73.1 | 97.2 | 132.5 | 190.4 | 88.9 | 39.5 |

^1^ Control diets (C) were formulated to meet nutrient requirement according to the standard Swiss feeding recommendations for grower finisher pigs in the respective growth periods; low protein diets (LP) were formulated to contain, expressed as percentage of the control diets, 80% of dietary CP, lysine, methionine + cystine, threonine and tryptophan.

^2^ New born piglets were not separated into the five different fractions. Results are presented in Supplementary Tables S4 and S5.

^3^ Skin and claws were not removed from the carcass of piglets slaughtered at 10 kg BW. Results of the amount of amino acids in the carcass of 10 kg BW pigs are available in Supplementary Table S4.
